# Higher Blood Pressure is Associated with Greater White Matter Lesions and Brain Atrophy: A Systematic Review with Meta-Analysis

**DOI:** 10.3390/jcm10040637

**Published:** 2021-02-07

**Authors:** Khawlah Alateeq, Erin I. Walsh, Nicolas Cherbuin

**Affiliations:** Centre for Research on Ageing, Health and Wellbeing, The Australian National University, Canberra, ACT 2601, Australia; erin.walsh@anu.edu.au (E.I.W.); nicolas.cherbuin@anu.edu.au (N.C.)

**Keywords:** blood pressure, white matter lesions, total brain, hippocampus, magnetic resonance imaging

## Abstract

Background: To summarise and quantify the evidence on the association between Blood pressure (BP), white matter lesions (WMLs), and brain volumes. Method: Electronic databases PubMed, Scopus, and Clarivate were searched in February 2020 using an established methodology and pre-determined search terms. Studies were eligible for inclusion if they reported on the association between BP and WMLs or brain volume in cognitively healthy individuals, while adjusting for age and intra-cranial volume. Results: Searches yielded 7509 articles, of which 52 (26 longitudinal and 33 cross-sectional), were eligible and had a combined sample size of 343,794 individuals. Analyses found that 93.7% of studies reported that higher BP was associated with poorer cerebral health (higher WMLs and lower brain volumes). Meta-analysis of compatible results indicated a dose-dependent relationship with every one standard deviation increase in systolic BP (SBP) above 120 mmHg being associated with a 11.2% (95% CI 2.3, 19.9, *p* = 0.0128) increase in WMLs and −0.13% (95% CI −0.25, −0.023, *p* = 0.0183) smaller hippocampal volume. Conclusion: The association between BP and brain volumes appears across the full range of BP measurements and is not limited to hypertensive individuals. Higher BP in community-residing individuals is associated with poorer cerebral health.

## 1. Introduction

The world population is ageing. The proportion of people aged over 65 years currently represents 15% of the global population, and it is predicted to grow to 22% by 2050 [1]. As a consequence, more people are expected to work and contribute to their communities for longer. However, for this to be possible, ageing individuals need to remain physically and cognitively fit. It is, thus, important to identify the risk factors for premature ageing so preventative actions can be implemented. A large body of evidence demonstrating a link between cardiovascular and physical health exists [2]. However, the association between cardiovascular health and brain health has received less attention, particularly in those who are not clinically impaired, and needs to be more precisely characterised.

Hypertension is a major risk factor for cerebral health. Midlife hypertension is associated with a two- to five-fold increased risk of stroke [3], and up to 50% greater risk of developing vascular dementia [4]. Moreover, hypertension is also linked to the development of amyloid angiopathy, the progression of white matter lesions (WMLs), and a reduction in global [5] and regional brain volumes [6]. Hippocampal atrophy, in particular, has been consistently reported in individuals with hypertension. This is significant as the hippocampal region plays a fundamental role in memory and overall cognition [7].

In recent times, increasing research has demonstrated that, not only hypertension, but elevated BP in the pre-hypertensive or even the upper normal range, may be detrimental to cerebral health [8]. However, the extent to which variation in BP across its full range impairs cerebral health is not fully understood. It has been long known that BP rises steadily with increasing age from early adulthood into older age [9]. Therefore, even small harmful effects experienced over decades could lead to a substantial deterioration of cerebral health. The importance of BP for cerebral health has also been acknowledged in a recent communication from the American Heart Association, which indicated that hypertension-related symptomatic clinical conditions, including cognitive dysfunction, could be avoided through primary prevention of BP elevations [10]. Consequently, it is important to develop a better understanding of the progressive impact of rising BP levels and brain structure and function, which will help promote and justify prevention earlier in life rather than in mid-life when hypertension typically develops. 

To address this gap, this systematic review aims to summarise and quantify the evidence of the association between BP, WMLs, and total and regional brain volumes as indexes of cerebral health in individuals free from cognitive impairment. A second aim is to investigate whether a dose-effect exists in the relationship between BP and brain volumetrics.

## 2. Methods

This review followed the Preferred Reporting Items for Systematic Reviews and Meta-Analyses (PRISMA), and was registered in the International Prospective Register of Systematic Reviews (PROSPERO, CRD42019123148) [11].

### 2.1. Search Strategy

PubMed, Clarivate Analytics, and Scopus were searched on with the following search string: (brain OR cerebral OR “white matter” OR “gray matter” OR hippocamp* OR amygdala) AND (volume* OR structu* OR thickness OR shrink* OR atrophy) AND (blood pressure OR BP OR hypertens* OR Prehypertens* OR normotens* OR systolic* OR diastolic* OR “pulse pressure” OR “arterial pressure”) AND(magnetic resonance imaging OR MRI OR neuroimaging OR image). Database filters were used to exclude studies written in a language other than English. 

### 2.2. Screening

Following previously established methodology [12], search results were first screened by title by one reviewer (KA). The abstracts of the remaining entries were double-screened (KA, NC and ER) against selection criteria. Any disagreement was resolved by consensus. The full text of selected studies and Supplementary Material were obtained and double-screened against inclusion and exclusion criteria. The reference lists of included studies and related reviews as well as gray literature were searched to identify additional studies which may not have been detected through the database searches. 

### 2.3. Selection Criteria and Study Screening

Studies were included based on the following criteria: (1) Recruitment adult human samples; (2) reported on volumetric measures of global and/or regional brain structure volumes derived from structural magnetic resonance imaging (MRI) brain scans; (3) reported on objective central or peripheral BP measures; (4) reported an association between BP measures and brain volumes; (5) included samples comprising generally healthy participants from the general population or from case-control studies (healthy controls); (6) adjusted for individual differences in head size and age; (7) used cross-sectional and/or longitudinal designs; and (8) interventional studies that report on a non-clinical (except for hypertension) control group.

Exclusion criteria were: (1) Exclusive focus on clinical/pathological populations; (2) no exclusion of neurological disorders (e.g., dementia); (3) inclusion of exclusively hypertensive populations, (4) case studies, theses, book chapters, author responses, conference papers, posters, reviews, non-peer reviewed publications, published abstracts or any other reports without full text; (5) samples with less than 40 participants to avoid sample bias and ensure only research of high quality is considered and; (6) non-English-language publications; (7) animal studies; and (8) post-mortem studies. 

### 2.4. Data Extraction

Data extraction was carried out by KA and checked by EW and NC. Any discrepancies in the extraction results were resolved by consensus. Information extracted included (1) basic study information (e.g., study title, author name, publication year); (2) study design and participants’ demographic characteristics (e.g., sample size, sample source, type of study, age, gender, co-variables, follow-up period); (3) neuroimaging technique (e.g., magnetic field, brain volumes, segmentation); (4) BP measurements (e.g., BP measuring technique, BP type, BP assessment); and (5) statistical information reporting a correlation between the differences in BP and the differences in brain volume (statistical method used for analysis, sample size, standardized and unstandardized β-coefficients efficient estimate, confidence interval [CI], standard error [SE], and *p*-value). If no SE value was reported, the SE was calculated from the CI or *p*-value [13]. Where required data were not reported in the included studies, authors were contacted for further information according to the PRISMA guidelines. Where missing data could not be sourced, the study contributed to the qualitative review but not to the meta-analysis.

### 2.5. Exposure

Non-invasive central BP was defined as central pulse pressure (CPP) measured based on carotid pressure waveforms calibrated using diastolic and integrated mean brachial pressure [13]. Peripheral BP was explored in terms of diastolic BP (DBP), systolic BP (SBP), pulse pressure (PP), and mean arterial pressure (MAP = DBP + (1/3 × (SBP − DBP).

### 2.6. Outcome Measure

Standardized and unstandardised β-coefficients from general linear models were considered as measures of association between BP and brain volume. All analyses were adjusted for basic covariates i.e., ICV and age. All types of brain volumes measures assessed with MRI were acceptable irrespective of the method used to measure them (manual or automated). For automated measures, the type of package used to extract them [14]. Standardized β-coefficients and the corresponding SE were pooled using random effects models, since studies were heterogeneous in design and methodology [15]. The heterogeneity across studies was assessed using the I^2^ statistic (values of 25%, 50%, and 75% are indicative of low, medium, and high heterogeneity, respectively) [15]. To assess the publication bias, we visually inspected the funnel plot for each brain volume outcome using ‘trim and fill’ methods [16]. All analyses were performed with R v.3.1 using the Metafor package 1.9-9 [17].

### 2.7. Meta-Analysis

Meta-analyses were conducted separately for SBP, DBP, PP and MAP, where a minimum of three studies was available. Studies were considered compatible for combined analysis if; (1) they provided an estimate for the same BP type (SBP, DBP, PP, or MAP) and reported an association with the same brain region; (2) the volumetric measure was the same across studies or could be transformed; (3) the same BP assessment methods was used (e.g., occasional, exercise, stress BP measure, or BP variability over different time points); (4) beta estimates for the same type of continuous BP measure were provided; (5) a similar study design (longitudinal or cross-sectional) was used; and (6) studies did not report on the same cohort. Therefore, only estimates from studies with the same study design that investigated the association between the same BP measures, as well as the same brain measures, and which reported compatible statistics, were combined. Where studies reported multiple association between BP and brain volumes with various types of BP measures (SBP, DBP, MAP, PP), or at different time points (i.e., cross-sectional, and longitudinal), each association was then investigated in separate analyses and results were not combined or averaged across BP types. Where multiple studies reported on the same cohort, the study with the largest sample was included. The effect of moderators including age, proportion of females, and hypertension was investigated by meta-regression. Analyses were performed using the Meta package 4.13-0 [18].

### 2.8. Quality Assessment

The studies were evaluated for methodological quality using an adapted Newcastle-Ottawa scale (Supplementary Information; Table S1).

## 3. Results

**Figure 1:**
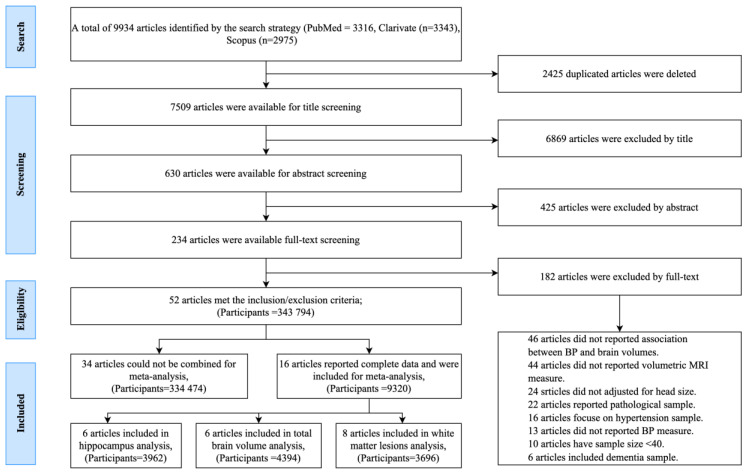
PRISMA flow diagram presenting the number of studies excluded and included at every stage of the review process, and the final number of studies included in the systematic review and meta-analysis.

The screening and study selection processes are presented in the PRISMA flow diagram (Figure 1). Fifty-two studies (*n* = 343,794, weighted mean age (MWA) = 58.7 years, women = 53.2%) met the inclusion criteria presented in Supplementary Information; Table S2. They included 33 cross-sectional [5,6,19,20,21,22,23,24,25,26,27,28,29,30,31,32,33,34,35,36,37,38,39,40,41,42,43,44,45,46,47] and 26 longitudinal studies [5,28,39,41,45,48,49,50,51,52,53,54,55,56,57,58,59,60,61,62,63,64,65,66,67] with follow-up ranging from ~2 [39,45,61] to 25 years [62] (six with both baseline and follow-up data) [5,28,39,41,45,48] surveying cognitively healthy participants. one study recruited only men [51], in eight studies [31,41,44,45,46,47,65,68] more than half of the participants were men, while two studies recruited only women [34,48] Participants ranged in age from 18 [34] to over 90 years [56]. Sample sizes ranged from 40 [21] to 4659 [32]. 

### 3.1. BP Assessment

Fifty-two studies reported on the peripheral BP measurement and one study additionally reported on non-invasive CPP measurement [60]. Typically, studies reported BP as a continuous measure (87.7%), but a small proportion (12.3%) reported a categorical measure (normotension vs. hypertension) [23,25,28,32,38,46,54,69] Several methods were used to assess BP including occasional BP (78.8%), 24-h ambulatory BP monitoring (ABP, 11.5%) [21,39,56,61,66,67], BP variability over different visits (9.6%) [26,57,62,63], BP during exercise (2%) [58], BP reactivity in response to stress (2%) [34], and inter-arm differences in SBP (2%) [27].

Overall 43.6% of participants were hypertensive (range 11–86.6%) [30,63]. Hypertension was defined based on; (1) use of anti-hypertensive medication (*n* = 36); (2) SBP and/or DBP cut-off values (*n* = 45); and (3) both cut-off values and anti-hypertensive medication (*n* = 37). Hypertension status was not reported in 13% of studies (Supplementary Materials). 

### 3.2. Magnetic Resonance Imaging

Most studies used magnetic field strengths of 1.5 Tesla (65.11%); others used 3 Tesla (14%), 4 Tesla (2.3%), 1 Tesla (2.3%), and 0.5 Tesla (2.3%), while 14% of studies did not report the magnetic field strength. Different brain segmentation methods were used, including manual tracing (20%), semi-automated segmentation (46.7%), and voxel-based morphometry (20%). However, the segmentation method used was not reported in 13.3% of studies. All studies included in the systematic review were adjusted to account for variation in head size, either in the statistical model or during image processing, by normalization against intra-cranial volume (ICV, 92.3%), average head size (3.8%) [42,44], or skull size (3.8%) [47,57] (Supplementary Information; Table S2).

### 3.3. Quality Assessment

**Figure 2:**
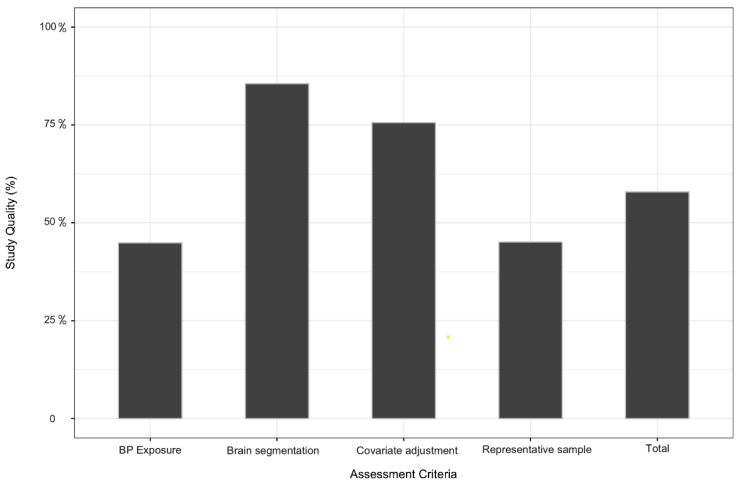
Quality rating of selected studies based on the adapted Newcastle-Ottawa Scale.

Most studies were rated as being of moderate (42.3%), or high (40.3%) quality, with only (17.3%) rated as low quality. The main weakness observed were in the assessment of BP exposure (44.9%), which included reporting the BP assessment protocol and defining hypertension criteria. This followed by the selection a sample not representative of the population they were drawn from (45.1%). Most studies (87.6%) were assessed the brain volumes blind to the BP level. Only few studies reported incomplete data on the outcome segmentation methods (Figure 2, Supplementary Information; Table S3).

### 3.4. Publication Bias and Heterogeneity

The trim and fill method was used to assess publication bias. The missing studies ranged from 0 to 2, representing approximately 16% (range 0–40%) of the studies included in the analyses. This suggests a relatively low level of bias. For most of analyses heterogeneity was moderate to high, although it was very low in HCV analyses (Supplementary Information; Figures S1–S10).

### 3.5. Association between Peripheral BP and Global and Regional Brain Volume

**Figure 3:**
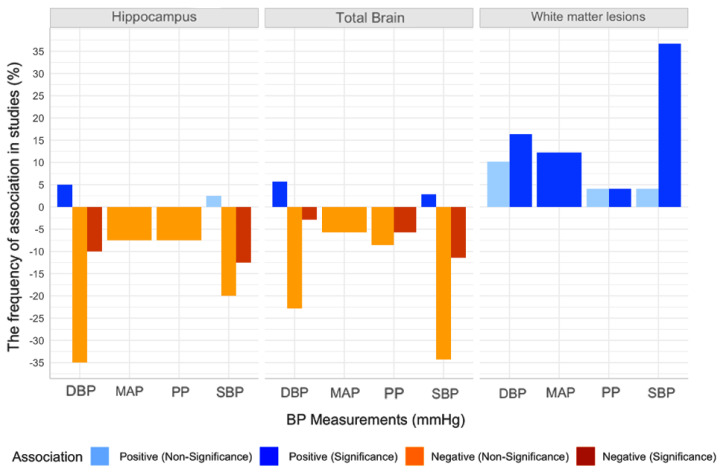
Proportion of the studies included in the systematic review (*n* = 52; sample size, *n* = 343,794), that reported an association between blood pressure (BP) and volumes of different brain regions. Notes. Where studies reported on different types of BP measurements, different measures were included in separate analyses. Systolic BP (SBP), diastolic BP (DBP), pulse pressure (PP), and mean arterial pressure (MAP).

Figure 3 shows the number of studies reporting negative and positive association between peripheral BP as a continuous measure and WMLs, or brain volumes, including total brain volume (TBV), and hippocampus volume (HCV). The majority of studies (93.7%) found that higher BP was associated with poorer cerebral health (either higher WMLs or lower brain volumes), although these associations only reached significance in (58.2%) of studies.

#### 3.5.1. BP and White Matter Lesions

##### Positive Association with White Matter Lesions

**Figure 4:**
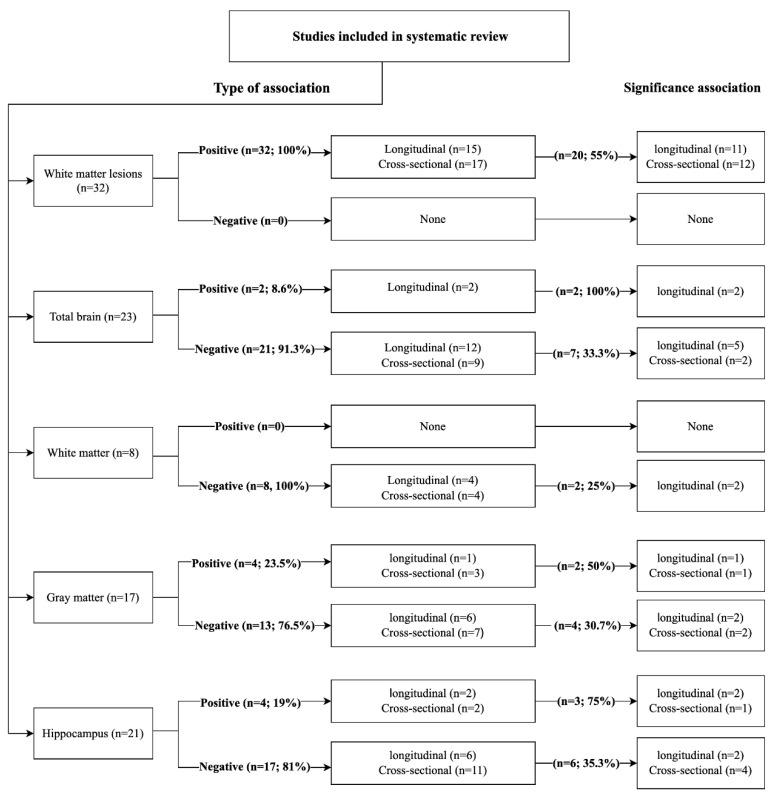
Flow diagram detailing the number of all included studies (*n* = 52) which reported positive or negative associations between BP and different brain regions.

Of the 52 studies included, 32 studies investigated the association between BP and WMLs volume, with all of them reporting a positive association (*n* = 17,472, MWA = 57.8 years, women = 55.1%, hypertension = 35.4%). Seventeen cross-sectional [4,19,22,24,28,30,35,36,37,38,40,41,42,44,45,46] and 15 longitudinal studies [4,19,38,40,44,49,52,53,54,55,59,60,62,66] found that higher BP (SBP, *n* = 48%; DBP, *n* = 31.2%; PP, *n* = 8.3%; MAP, *n* = 12.5%) was associated with larger WMLs. The relationship reached significance in 20 (62.5%) studies (82.6% of participants; 55% longitudinal) (Figure 4) [4,19,24,28,35,36,37,38,40,42,44,46,51,52,53,55,59,60,62,66].

##### Negative Association with WMLs

None of the studies reported a negative association with WMLs (Figure 4).

##### Meta-Analysis of BP and WMLs

**Figure 5:**
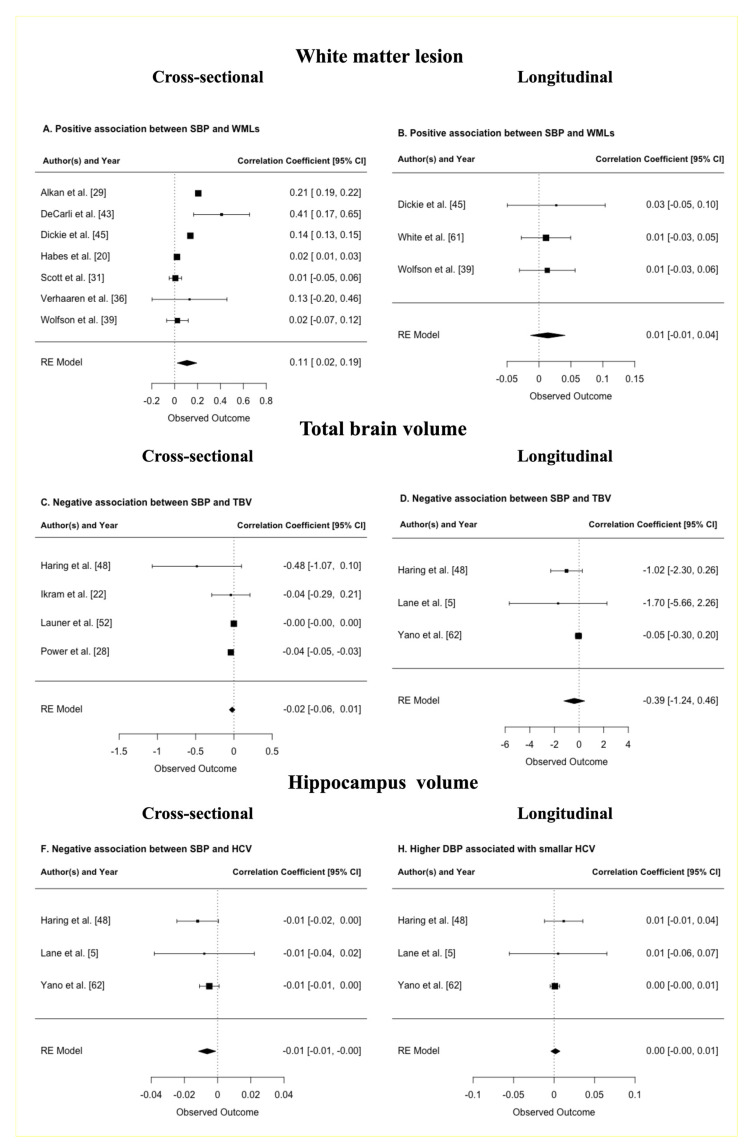
Forest plots of analyses investigating the association between continuous systolic blood pressure (SBP) and brain volumes in cm^3^; (**A**) shows a positive association between SBP and white matter lesions (WMLs) in cross-sectional studies; (**B**) shows a positive association between SBP and WMLs in longitudinal studies; (**C**) shows a negative association between SBP and total brain volume (TBV) in cross-sectional studies; (**D**) shows a negative association between SBP and TBV in longitudinal studies; (**E**) shows a negative association between SBP and smaller hippocampus volume (HCV) in cross-sectional studies; and (**F**) shows a negative association between SBP and HCV in longitudinal studies. The effect size is a standardized beta-coefficients. The sizes of the squares reflect the weight given to each effect size.

Eight studies provided data on WMLs that could be pooled (*n* = 3696, MWA = 58.2 years, women = 45.5%, hypertension = 33.1%) (Table 1). The meta-analysis of seven cross-sectional studies indicated that every one standard deviation (SD) increase in SBP above 120 mmHg was associated with 108 mm^3^ (95% CI 23, 193, *p* = 0.0128) larger WMLs (Figure 5A) [20,29,31,36,39,43,45]. No association was detected across three longitudinal studies with a mean weighted length of follow-up of 2 years. All analyses were adjusted for ICV, and age (Figure 5B) (Supplementary Information; Figures S1–S3) [39,45,52,61].

A sensitivity analysis was conducted to investigate whether the association between SBP and WMLs volume differs between younger and older individuals [20,29,31,36,39,43,45]. Since most studies reported a mean age above ~60 years, the available studies were stratified based on a sample mean age + 2 SD falling above or below 75 years. Results indicate that the effects are consistent below and above our threshold. However, in younger individuals (mean weighted age ~72 years) the effect was stronger. In contrast, while still significant in older individuals (mean weighted age 80.6 years) the effect was much reduced (Supplementary Information; Figure S12). 

##### Meta-Regression of BP and WMLs

The moderating effects of mean age (range 52–81 years), and the proportion of female (range 57–61%) or hypertension (range 23.9–69%) on the association between BP and WMLs was investigated by meta-regression. None of the effects reached significance [20,29,31,36,39,43,45].

#### 3.5.2. BP and Total Brain Volume

##### Positive Association with TBV

Twenty-three studies investigated the relationship between BP and total brain volume (TBV), with two (8.6%) studies (34.2% of participants) reporting a significant positive association (*n* = 8019, MWA = 54.9 years, women = 56.7%, hypertension = 23.4%). Two longitudinal studies showed that BP (SBP, *n* = 1; DBP *n* = 2) was positively associated with smaller TBV (Figure 4) [49,53].

##### Negative Association with TBV

Of the 23 studies, 21 (91.3%) studies (67.8%of participants) found a negative association between BP and TBV (*n* = 15,368, MWA = 57.1 years, women = 55.8%, hypertension = 26.5%). Nine cross-sectional [5,22,25,27,28,32,38,41,47] and 12 longitudinal studies [5,28,41,48,49,54,55,58,60,62,66,67] found that higher BP (SBP, *n* = 50%; DBP, *n* = 28%; MAP, *n* = 6.2%; PP, *n* = 15.6%) was associated with smaller TBV. The relationship reached significance in seven (33.3%) studies (26.9% of participants; 71.4% longitudinal studies) (Figure 4) [27,38,54,55,58,62,67].

##### Meta-Analysis of BP and TBV

Six studies provided data on TBV that could be pooled (*n* = 4394, MWA = 54.7 years, women = 61.1%, hypertension = 37.1%) (Table 1). The meta-analysis of three longitudinal studies indicated that every one-SD increase in SBP and DBP (changes) was associated with a 386 mm^3^ (95% CI −123, 464, *p* = 0.3738), and 490 mm^3^ (95% CI −729, 6310, *p* = 0.8877), smaller TBV respectively, although these associations did not reach significance (Figure 5D) [5,48,62]. No association was detected across four cross-sectional studies. All analysis controlled for head size, age, and sex (Figure 5C, Supplementary Information; Figures S4–S7) [22,25,28,48].

#### 3.5.3. BP and White Matter Volume

##### Positive Association with WM Volume

None of eight studies investigating the relationship between BP and white matter (WM) volume found a positive association [22,32,33,53,54,57,62,64].

##### Negative Association with WM Volume

All of the eight studies found a negative association between BP measures and WM volume (*n* = 10,925, MWA = 54.5 years, women = 53.2%, hypertension = 33.7%). Four cross-sectional [22,32,33,64] and four longitudinal studies [53,54,57,62] found that higher BP (SBP, *n* = 40%; DBP, *n* = 53.3%; PP, *n* = 6.6%) was associated with smaller WM. The relationship reached significance in only two (25%) of the longitudinal of the studies (17.3% of participants) (Figure 4) [33,57,62].

#### 3.5.4. BP and Grey Matter (GM) Volume

##### Positive Association with GM Volume

Seventeen studies investigated the relationship between BP and grey matter (GM) volume, with four (23.5%) studies (32.2% of participants) reporting a positive association (*n* = 4755, MWA = 53.3 years, women = 59%, hypertension = 32.6%). Three cross-sectional [6,22,23] and one longitudinal studies [53] showed that BP (SBP, *n* = 33.3%; DBP, *n* = 50%; MAP, *n* = 16.6%) was positively associated with GM. The relationship reached significance in two (50%) of studies, (88.3% of participants; 50% longitudinal) (Figure 4) [5,21].

##### Negative Association with GM Volume

Out of 17 studies, 13 (76.5%) studies (67.8% of participants) found a negative relationship between BP and GM (*n* = 9996, MWA = 51.6 years, women = 51.9%, hypertension = 30%). Seven cross-sectional [6,23,24,30,32,33,64] and six longitudinal studies [53,54,57,59,62,65] found that higher BP (SBP, *n* = 40.4%; DBP, *n* = 53.3%; PP, *n* = 5%; MAP *n* = 10%) was associated with smaller GM. The relationship reached significance in four (30.7%) studies (24.4% of participants; 50% longitudinal studies) (Figure 4) [6,30,54,62]. 

When testing the association between BP and regional brain volumes, a negative association was reported in the amygdala [27,44], insula [34,67], basal ganglia [70], and thalamus as well as in the medial temporal, frontal [70], and parietal lobe structures [30].

#### 3.5.5. BP and Hippocampal Volume

##### Positive Association with HCV

Twenty-one studies investigated the relationship between BP and hippocampal volume (HCV), with four (19%) studies (22.3% of participants) reporting a positive association (*n* = 1814, MWA = 71 years, women = 33.5% hypertension = 28.4%). Two cross-sectional studies [26,44] surveying the same cohort and two longitudinal studies [48,64] found that BP (SBP, *n* = 1; DBP, *n* = 2) was positively associated with HCV. The relationship was significant in three (75%) studies (26.1% of participants; 66.6% longitudinal) (Figure 4) [44,48,64].

##### Negative Association with HCV

Out of 21 studies, 17 (81%) studies (77.7% of participants) found a negative association between BP and HCV (*n* = 6317, MWA = 59 years, women = 46.4%, hypertension = 30.3%). Eleven cross-sectional [5,19,26,28,30,34,38,40,41,44,47] and six longitudinal studies [28,41,51,57,62,71] found that higher BP (SBP, *n* = 35.1%; DBP, *n* = 48.6%; MAP, *n* = 8.1%; PP, *n* = 8.1%) was associated with smaller HCV. The relationship was significant in six (35.3%) of studies (28% of participants; 42.8% longitudinal) (Figure 4) [26,30,34,44,57,62].

##### Meta-Analysis of BP and HCV

Six studies provided data on HCV that could be pooled (*n* = 3962, MWA = 56.1 years, women = 60.8%, hypertension = 34.7%) (Table 1). The meta-analysis of three longitudinal studies with a mean weighted length of follow-up of 11.13 years indicates that every one-SD increase in SBP above 120 mmHg, and DBP was associated with a −6.3 mm^3^ (95% CI −11.6, −1.1, *p* = 0.0183) and 1.7 mm^3^ (95% CI −4, 7, *p* = 0.5666) smaller HCV (Figure 5F) [5,34,40]. No association was detected across three cross-sectional and four longitudinal studies with SBP [26,28,48] or DBP [26,28,44,48] respectively. All analyses were controlled for ICV, age, and sex (Figure 5E, Supplementary Information; Figures S8–S11).

### 3.6. Association between Centeral BP and Global and Regional Brain Volume

One study reported on the association between central BP and cerebral health (*n* = 1223, mean age = 61 ± 9 years, 56% women, hypertension = 28%). No significant associations between CPP and WMLs (*p* = 0.74) or TBV (*p* = 0.95) over 6.4 ± 1.3 years of follow-up was detected [60]. 

## 4. Discussion

This study systematically reviewed the existing literature on the association between BP and brain volumes. Overall, it confirmed that increased BP is a substantial risk to ce-rebral health. The review produced several important findings. Firstly, it demonstrated that the associations between BP and brain volumes emerge across the full range of BP measurements and are not limited to hypertensive individuals. Secondly, the associations between BP and brain volumes were found to be dose-dependent. Thirdly, effects were strongest for the hippocampus and WMLs, but mostly reflected the results of cross-sectional studies. In contrast, analyses based on relatively sparse longitudinal data demonstrated weaker associations.

The key finding of this systematic review is that the vast majority of articles (93.7% of the 52 articles included in the review) found that higher BP was associated with poorer brain health. The effect of BP varied across brain regions, but consistent evidence suggested particularly strong associations for WMLs and the hippocampus. The magnitude of these effects were large and dose-dependent, with every one-SD higher SBP being associated with an 11.2% larger WMLs volume in cross-sectional studies. However, the association between SBP and WMLs was substantially weaker in longitudinal analyses, which were based on a very small number of studies. Consistent with these findings, similar associations, albeit weaker, were also found in relation to TBV with 91.3% of studies reporting higher BP to be associated with lower volume. This effect may have been substantially driven by smaller white matter and higher WMLs, as consistent associations between BP and these measures were observed across all studies.

Since the existing literature is inconclusive on whether SBP or DBP has a greater impact on brain health, we contrasted the effect of SBP and DBP on HCV. We found that higher SBP was more strongly associated with lower volume than DBP, but only in the hippocampus. This may suggest that SBP has a somewhat greater impact on brain health. However, it must be noted that the studies included in this analysis mostly consisted of individuals who were on average in their mid-fifties, which may indicate that this difference might be due to changes in SBP and DBP patterns at this point in life, as DBP tends to decline after the age of 50–60, while SBP continues to increase with age [71]. 

These findings have significant clinical implications since TBV, but particularly HCV, are implicated in the onset and progression of Alzheimer’s disease (AD). HCV is strongly predictive of conversion to AD and therefore, any additional shrinkage in this brain regions attributable to higher BP is likely to hasten conversion. Hippocampal shrinkage in normal aging is estimated to be slightly over 1% per/year above 70 [72], and twice this amount in the pre-clinical stage of the disease [73]. Thus, we estimate that the additional 2.6% shrinkage experienced by somebody with hypertension (SBP = 140 mmHg), compared to somebody with normal SBP (120 mmHg), might lead to premature AD conversion by a year or more [73]. Consequently, since the mean age of the samples included in the meta-analysis was 52 years and above, it is critical that prevention efforts be directed at younger adults, not only to protect brain health in general, but also to decrease future risk of developing dementia.

Another important finding was that increased BP was associated with a poorer brain health across its full range, and not exclusively in individuals with hypertension or pre-hypertension. Indeed, the meta-regression testing the effect of the proportion of participants with hypertension across different studies, which ranged from 23.9% to 69%, revealed no significant effect of hypertension on brain health. This indicates that associations between BP and brain measures are not mainly driven by those individuals with hypertension and further emphasizes the need for risk reduction before hypertension develops. Additionally, these findings suggest that more systematic BP and overall health monitoring, as well as the promotion of a healthier lifestyle, should be implemented at a younger age and supported through educational campaigns.

The pathological mechanisms linking BP to overall and localized brain atrophy and cognitive decline are not fully understood. Several mechanisms, including neuroinflammation, oxidative stress, dendritic shrinkage, and apoptosis, are thought to be implicated in the pathophysiology linking elevated BP and neurodegeneration. Indeed, higher BP levels have been shown to up-regulate the production of pro-inflammatory cytokines [74,75,76]. In turn, chronic systematic inflammation produces higher levels of oxidative stress, which leads to DNA damage and impairment of cellular structure and function [75]. Thus, through these mechanisms, elevated BP is likely to contribute to dendritic shrinkage, decreased neurogenesis, demylination, and neuronal loss [77] which are detectable at a macroscopic level as brain atrophy, particularly in the hippocampus.

In addition, the etiology of WMLs is of particular significance, as they impact cognitive function across all domains, and generally to a greater extent than brain atrophy [78]. While the pathophysiological mechanisms reviewed above are also implicated in the development of WMLs, cardio-vascular factors are thought to be the main contributors. Good evidence suggests that BP increases the risk of arthrosclerosis by 50% or more [79]. This is likely to lead to lower blood perfusion in capillaries, endothelia dysfunction [80], impaired vasoreactivity, increased pulsatility, vessel stiffening, and changes to the blood brain barrier (BBB) integrity. Resulting small vessel disease in conjunction with ischemia, inflammation, and myelin loss are then likely to contribute to the development of WMLs [81].

The progression of WMLs may also contributes to worse global and regional brain atrophy. Although the precise nature of this relationship is not fully clear, advanced neuroimaging methods suggest that WMLs particularly affect white matter networks connecting remote brain regions and thus lead to gray matter shrinkage, for example through Wallerian degeneration [82]. This makes it particularly important to assess an individual’s brain health profile with both WMLs and tissue loss, [78] so we can develop a better understanding of their inter-relationship and underlying pathological mechanisms [78].

A somewhat surprising result is that the association between BP and brain volume was, as demonstrated in the sensitivity analyses of WMLs, somewhat stronger in mid-life individuals although it remained significant into old age. The reasons for this effect are not completely clear but may be due to sample or study characteristics. Alternatively, it has been shown that vascular structure changes with advanced age. Therefore, it is possible that endothelial sensitivity to increasing BP varies across age groups [83,84]. In contrast, no moderating effect of sex was detected despite several previous reports suggesting differential effects of BP in men and women [6]. This may be due to the approximate nature of the sex analyses, which were based on the sex ratio of each sample, rather than on individual-centered data. Therefore, future studies should aim to report separate estimates for men and women so more precise syntheses can be conducted. 

Previous literature has determined that the progression and distribution of WMLs differ in individuals from European and Asian background, with European individuals tending to experience greater WMLs load [85]. It would have been of interest to address this question in the present review. Unfortunately, as most studies included consisted predominantly of people with a Caucasian background, it was not possible to investigate the impact of ethnicity in this study. To fill this gap, future studies should more consistently report the ethnic composition of their sample and, where possible, conduct stratified analyses to shed light on this important question. 

Finally, included studies substantially differed in their methodology and robustness as demonstrated by their quality rating. On average studies were rated as having moderately good quality. The greatest weakness identified related to BP exposure, with 44.9% of studies reporting incomplete information about hypertension level and failing to describe their BP measurement protocol. Another weakness stems from the fact that ~45% of studies did not select a representative sample of the general population. Furthermore, important variation in how different studies controlled for major covariates was also identified, and in some instances limited adjustment may have somewhat biased their findings. These considerations highlight the need for greater consensus and standardized methodology for epidemiological studies investigating BP correlates. 

This systematic review had a number of limitations. Firstly, while a moderately high number of studies were identified for inclusion, a relatively small number of studies which reported suitable statistics (β-coefficients) could be pooled and meta-analysed. Secondly, a majority of studies included samples with wide age ranges, which makes it difficult to separate the effects of BP from those associated with other ageing processes. Thirdly, most findings were based on cross-sectional results. The limited number of longitudinal analyses produced weak findings, which may reflect a lack of statistical power, or other sample or study characteristics. Finally, while the association between BP and WMLs is known to differ across ethnic background, insufficient ethnic data was available to investigate these effects [85]. Thus, future research should focus on better characterising the effect of higher BP in populations of diverse ethnic origins. This study also had many strengths. It included a very broad literature search using clearly defined search terms and stringent criteria for inclusion and exclusion. Particular care was taken to only include studies including participants with normal cognitive function, free of neurological disorders, and who were not recruited exclusively from a clinical population. Research that employed subjective ratings or did not adjust for age and head size was also excluded to minimize the impact of operator or sampling biases. 

## 5. Conclusions

Although reviews have been previously published in this area, they only investigated the effects of hypertension on brain volume [86]. To the best of our knowledge, this study is the first systematic review with meta-analysis providing quantitative evidence on the negative association between continuous BP and global and regional brain volumes. Our results suggest that heightened BP across its whole range is associated with poorer cerebral health which may place individuals at increased risk of premature cognitive decline and dementia. It is therefore important that more prevention efforts be directed at younger populations with a greater focus on achieving optimal BP rather than remaining below clinical or pre-clinical thresholds [5].

## Figures and Tables

**Table 1:** Characteristics all included studies which contributed to the meta-analysis (*n* = 16).

Author, and year	Study Setting/Design	N	Age M (SD)	Sex (% female)	BP Methods	SBP M (SD)	DBP M (SD)	%HT	%AHT	Brain Region	Magnet/Segmentation	Covariates	Meta-Analysis of:
Alkan et al. 2019 [29]	The Baependi Heart Study/Cross-sectional	164	60.1 (7.8)	59.1	Occasional	129.6 (16.9)	79.5 (19.2)	54.5	NR	WMLs	1.5 T/Semi-automated	Age, education, BMI, WC, cholesterol, FBG, triglyceride, HDL-C, LDL-C, SBP, DBP, effect of sex and number of MetS	SBP and WMLs (cross-sectional)
DeCarli et al. 1995 [43]	Cross-sectional/National Institute on Aging	51	52 (20)	49	Occasional	124 (14)	78 (9)	0	NR	WMLs	0.5 T/NR	Age and education	SBP and WMLs (cross-sectional)
Den Heijer et al. 2005 [44]	Rotterdam Study/Cross-sectional and Longitudinal	511	73.4 (8)	49.1	Occasional	145.8 (20.3)	76.5 (11.6)	NR	38.9	HCV, Amygdala	1.5 T/Manual	Age, sex and CVD factors	DBP and HCV (cross-sectional)
Dickie et al. 2016 [45]	Community dwelling/Cross-sectional and longitudinal (~2 years)	681	72.7 (0.7)	47	Occasional	146 (18)	79 (9)	48.2	NR	WMLs	1.5 T/Semi-automated	Sex, BMI, and CVD history	SBP and WMLs (cross-sectional and longitudinal)
Habes et al. 2016 [20]	SHIP study/Cross-sectional	2367	52.4 (13.7)	56.7	Occasional	127.3 (17.6)	NR	NR	32.7	WMLs	NR/Semi-automated	Age, sex and education	SBP and WMLs (cross-sectional)
Haring et al. 2019 [48]	Women’s Health Initiative/Longitudinal (~ 8 years)	558	78.3 (3.6)	100	Variability	122 (1)	73 (7)	48	NR	Regional GM	3 T/Semi-automated	Age, education, APOE4 allele	(SBP, DBP) and TBV/ (SBP, DBP) and HCV (longitudinal)
Ikram et al. 2008 [22]	Rotterdam study/Cross-sectional	490	73.4 (7.9)	50.8	Occasional	NR	NR	51	0	TBV, GM, WM	1.5 T/Manual: TR was blinded to information	Age and sex.	(SBP, DBP) and TBV (cross-sectional)
Lane et al. 2019 [5]	NSHD/Cross-sectional and Longitudinal	441	36	49	Occasional and changes	120.2 (13.7)	78.4 (9.5)	16	2	TBV, HCV WMLs,	automated 3 T/Semi-automated	Sex, APOE ε4 status, AHT medication, and BP at 69 years of age.	SBP, DBP) and TBV/(SBP, DBP) and HCV (Cross-sectional and Longitudinal)
		441	43	49	Occasional and changes	123.5 (13.7)	80 (9.3)	52	28				
		441	53	49	Occasional and changes	133.5 (19)	83.1 (11.8)	46	12				
		441	60-64	49	Occasional and changes	124.9 (16.9)	77.4 (9.5)	22	2				
		441	69	49	Occasional and changes	120.2 (13.7)	78.4 (9.5)	16	2				
Launer et al. 2015 [52]	CARDIA/Cross-sectional	680	50.3 (3.5)	52.2	Occasional	139.9 (1.5)	79.5 (0.9)	32.2	NR	TBV	3 T/Semi-automated	Age, sex, and race.	(SBP, DBP) and TBV (Cross-sectional)
McNeil et al. 2018 [26]	Aberdeen 1936 Birth Cohort/Cross-sectional	227	64.5 (0.8)	52	Occasional	139.9 (1.5)	79.5 (0.9)	NR	45	HCV	1.5 T/Semi-automated	Age, sex, and AHT medication	SBP, DBP and HCV (Cross-sectional)
Power et al. 2016 [28]	ARIC study/Cross-sectional and Longitudinal (~15 and ~24 years)	1678	52.0	61	Occasional	130 (5.9)	66 (3.6)	23.0	72.0	TBV, HCV, brain lobes	3 T/Semi-automated	Age, sex, race, education, ICV, BMI, DM, cholesterol, and smoking status	(SBP, DBP) and TBV /(SBP, DBP) and HCV (Cross-sectional and Longitudinal)
Scott et al. 2015 [31]	ADNI/Cross-sectional	150	73.7 (6.3)	48.7	Occasional	136 (16)	75 (10)	44.0	NR	WMLs	3 T/NR	Age	SBP and WMLs (Cross-sectional)
Verhaaren et al. 2013 [36]	Rotterdam Study/Cross-sectional and longitudinal	665	61.6 (5)	52	Occasional	138 (19)	78 (10)	25.9	22	WMLs	1.5 T/Semi-automated	Age, sex, and ICV, CVD factors	SBP and WMLs (Cross-sectional)
White et al. 2011 [61]	Community dwelling/Longitudinal (~2 years)	72	82.1 (3.9)	56.9	Occasional	122 (1.3)	73 (7)	70	64.0	WMLs	3 T/Semi-automated	Age and LDL cholesterol levels,	SBP and WMLs (longitudinal)
Wolfson et al. 2013 [39]	Community dwelling/Cross-sectional and Longitudinal (~2 years)	67	81.7 (3.9)	61.0	ASBP	138 (14)	69 (7)	NR	69.0	WMLs	3 T/Semi-automated	Age, sex, and BMI or education	SBP and WMLs (Cross-sectional and longitudinal)
Yano et al. 2017 [62]	CARDIA/Longi-tudinal (~2 years)	547	25.6 (3.4)	53.9	Variability	123.2 (12.2)	73.4 (8.5)	51.8	21.2	TBV, GM, WM, HCV	3 T/Semi-automated	Age, sex, ICV, AHT medications, education, fasting glucose, smoking, and physical activity and BMI	SBP and HCV (longitudinal)

M = mean; SD = standard deviation; SBP = systolic blood pressure; DBP = diastolic blood pressure; ASBP = ambulatory systolic blood pressure; ABP = ambulatory blood pressure; WMLs = white matter lesions; TBV = total brain volume; GM = grey matter; WM = white matter; HCV = hippocampal volume; ICV = intra-cranial volume; IPFC = lateral prefrontal cortex, pFWM = prefrontal white matter. CVD = cardiovascular disease; HT = Hypertension; ATH=antihypertensive; BMI = body mass index; DM = diabetes mellitus; WC = waist circumference, FBG = fasting blood glucose; APOE e4 = Apolipoprotein E; HDL-C = High-density lipoprotein cholesterol; LDL-C = low density lipoprotein-cholesterol, MetS = metabolic syndrome; SES = socioeconomic status; T = tesla; ADNI = The Alzheimer’s Disease Neuroimaging Initiative, ARIC = The Atherosclerosis Risk in Communities study; CARDIA = Coronary Artery Risk Development in Young Adults; NSHD = National Survey of Health and Development; HIP = Study of Health in Pomerania; TR = clinically trained.

## Data Availability

Data are available from the authors on request.

## Supplementary Materials

The following are available online at https://www.mdpi.com/2077-0383/10/4/637/s1. Table S1. Adjusted Newcastle-Ottawa Quality Assessment Scale for Studies, Table S2. Characteristics of the selected studies, Table S3. Methodological quality of studies, Figure S1. Association between SBP and white matter lesions from cross-sectional studies A. Forest plots; B. Sensitivity Analysis; trim and fill, Figure S2. Association between SBP and white matter lesions from longitudinal studies. A. Forest plots; B. Sensitivity Analysis; trim and fill, Figure S3. Association between DBP and white matter lesions from longitudinal studies. A. Forest plots; B. Sensitivity Analysis; trim and fill; Figure S4. Association between SBP and total brain volume from cross-sectional studies. A. Forest plots; B. Sensitivity Analysis; trim and fill; Figure S5. Association between DBP and total brain volume from cross-sectional studies. A. Forest plots; B. Sensitivity Analysis; trim and fill; Figure S6. Association between SBP variability and total brain volume from longitudinal studies. A. Forest plots; B. Sensitivity Analysis; trim and fill; Figure S7. Association between DBP variability and total brain volume from longitudinal studies. A. Forest plots; B. Sensitivity Analysis; trim and fill; Figure S8. Association between DBP and hippocampal volume from cross-sectional studies. A. Forest plots; B. Sensitivity Analysis; trim and fill; Figure S9. Association between DBP and hippocampal volume from cross-sectional studies. A. Forest plots; B. Sensitivity Analysis. Figure S10. Association between SBP variability and hippocampal volume from longitudinal studies. A. Forest plots; B. Sensitivity Analysis; trim and fill; Figure S11. Association between DBP variability and hippocampal volume from longitudinal studies. A. Forest plots; B. Sensitivity Analysis; trim and fill. Figure S12. The Forest plots show the association between SBP and white matter lesions in elderly below or above ~75 years. Given the small number of studies these results should be interpreted with caution. However, the pattern of results appears to indicate that effects are consistent below in younger individuals (mean weighted age ~72 years). In contrast, while still significant in older individuals (mean weighted age 80.6 years) the effect appears much reduced in this age group.
