# Supplementary material for: Higher Blood Pressure is Associated with Greater White Matter Lesions and Brain Atrophy: A Systematic Review with Meta-Analysis
Source: J Clin Med. 2021 Feb 7;10(4):637. doi: 10.3390/jcm10040637 (PMC7915964; doi:10.3390/jcm10040637)
Supplement: Supplementary file 1 [file jcm-10-00637-s001.pdf]

Journal of
Clinical Medicine

MDPI

Supplementary information

Higher blood pressure is associated with greater white matter lesions and brain atrophy: a systematic review with meta-analysis acronym defined

Khawlah Alateeq, Erin Walsh, Prof. Nicolas Cherbuin

Appendix A. Supplemental results

1. Magnetic resonance imaging

All studies included in the systematic review were adjusted to account for variation in head size, either in the statistical model or during image processing, by normalization against intracranial volume (ICV, 46 studies), average head size (two studies) [1,2], or skull size (two studies) [3,4].

2. BP assessment

Outcome measures included SBP (45%), DBP (39%), PP (8%) and MAP (6.9%). Apart from BP cut-off values, some studies used different values, namely i.e. ≥140/90,(n=) 150/90 (*n* = 1) [5] 160/95 (*n* = 1) [2] 160/100 (*n* = 2) [6,7] or 160-179/90-99 (*n* = 1) [8] as clinical measures and 135/85 for ABP [9]. Hypertensive participants were below 25% in (29.8% of studies), between 26% to 50% in (49% of studies), and above 50% in (21% of studies).

3. BP brain volumes and Age

3.1. BP associations in young adults

Five studies reported association between BP and brain volumes in young adults (18-40 years) [10–14]. Higher BP (SBP, *n* = 15; DBP, *n* = 12) was associated with greater (WMLs, *n* = 2[13,14]) and smaller brain volumes (TBV, *n* = 3 [10,13,14]; GMV, *n* = 2 [10,12]; WMV, *n* = 1[10]; HCV, *n* = 4[10–13]; amygdala, *n* = 1[11]; Insula, *n* = 2[11]). None of the association was significant in young adults.

3.2. Brain volumes in middle age

Twelve studies reported association between BP and brain volumes in middle-aged adults (50-60 years) [13,15–25]. Higher BP was (SBP, *n* = 17%; DBP, *n* = 9%; MAP, *n* = 7%; PP, *n* = 6%) was associated with larger WMLS (*n* = 6) [13,15,17–19], and smaller brain volumes including (TBV, *n* = 6 [13,17,18,20,21,26]; GMV, *n* = 1[26]; WMV, *n* = 1[26]; HCVs, *n* = 2) [13,17].

3.3. BP associations in older age

Fifteen studies reported association between BP and brain volumes in older adults (≥ 70 years). [2,4,5,13,16,17,27–35]. Higher BP was associated with larger WMLSCV (*n*= 8) [5,17,27,28,32–35]. Lower BP (DBP, *n* = 3; SBP, *n* = 1) was associated with smaller TBV (*n* = 1) [30] HCV (*n* = 2) [2,29] Higher SBP was associated with larger HCV [16]. However, higher BP was associated with smaller TBV (*n* = 2 [13,30]; HCV, *n* = 6[13,30,31]).

Table S1. Adjusted Newcastle-Ottawa Quality Assessment Scale for Studies.

Criteria	Rating	Stars
**Selection**		1 ☆

1.Representative of the general population	i. Generally representative	☆
	ii. Somewhat representative	½☆
	iii. Selected group	0
	iv. No description of the derivation of the cohort	0
**Exposure (BP)**		7 ☆
1. location of BP Measurement is reported	i. Location e.g. upper arm, ankle, or central	☆
	ii. Not stated	0
2. Position when BP Measurement parameters are reported	i. Position/posture e.g. standing, sitting, or supine	☆
	ii. Not stated	0
3. Resting period before BP measurement is reported	i. Resting period ≥ 3 min	☆
	ii. Resting period < 3 min	0
	iii. No resting period before BP measurement	0
	iv. Not stated	0
4. Number of BP readings is reported	i. Number of BP readings ≥ 2	☆
	ii. Number of BP readings < 2	0
	iii. No description	0
5. Time intervals between BP readings is reported	i. Time intervals between readings ≥1 min	☆
	ii. Time intervals between readings < 1 min	0
	iii. No description	0
6. Hypertension was defined by two criteria	i. BP level	½☆
	ii. Anti-hypertensive medication	½☆
	iii. No stated	0
7. Number of BP assessment sessions	i. Average of BP sampling over a day or longer e.g. ambulatory BP	☆
	ii. Average of multiple BP measurements taken over a day or longer e.g., BP variability	☆
	iii. Repeated occasional BP measures over time	½☆
	iv. Occasional BP	0
	v. No stated	0
**Comparability (confounder)**		1½☆
1. Confounders controlled in analyses	i. Analyses control for age and sex	☆
	ii. Analyses control for additional confounders	½☆
	iii. Not stated	0
**Outcome**		1☆
1. Measurement of brain volume/segmentation	i. Measurement conducted without knowledge of the exposure (e.g. fully automated segmentation)	☆
	ii. Measurement conducted without knowledge of the exposure (e.g. semiautomated segmentation)	☆
	iii. Measurement done with knowledge of the exposure (Manual)	0
	iv. No description	0

Table S2. Characteristics of the selected studies.

Study	Study Design	N	Age M (SD)	Sex (% female)	BP Methods	SBP M (SD)	DBP M (SD)	%HT	%AHT	Brain Region	Magnet / Segmentation	Covariables
Alkan et al 2019[36]	Cross-sectional	164	60.1 (7.8)	59.1	Occasional	129.6 (16.9)	79.5 (19.2)	54.5	NR	WMLS	1.5 T/ Semi-automated	Age, education, BMI, WC, cholesterol, FBG, triglyceride, HDL-C, LDL-C, SBP, DBP, and number of MetS
Allan et al 2015[17]	Cross-sectional and Longitudinal	190	69.3 (5.4)	18.4	Occasional	152.6 (1.3)	82.4 (1.1)	52.2	NR	WMLS, TBV, HCV	3 T/ Semi-automated	Age and sex
Bender et al 2012[25]	Cross-sectional	22<br>50	49.0 (17.3)<br>50.4 (12.9)	0<br>100	Occasional	122.3 (10.2)<br>119.6 (12.8)	75.6 (8.6)<br>73.1 (6.5)	0	0	HCV, IPFC, pFWM	4 T/ Manual	NR
Brickman et al 2010[27]	Longitudinal	176<br>166<br>167<br>177	79.3 (5.4)<br>80.8 (5.6)<br>80 (5.3)<br>80 (5.6)	73.3<br>69.3<br>61.7<br>65.5	Variability	130.3 (11.0)<br>131.3 (10.2)<br>151.8 (12.8)<br>155.9 (14.7)	68.2 (5.9)<br>68.6 (5.5)<br>79.8 (5.6)<br>80.9 (6.6)	0<br>0<br>100<br>100	57.9<br>68.4<br>65.5<br>73.2	WMLS	1.5 T/ Manual	Age, sex, treatment status
Burns et al 2005[27]	Cross-sectional	88	76.9 (8.2)	70.5	Occasional	135.8 (19.2)	72.2 (10.3)	39.8	NR	WMLS	1.5 T/ Semi-automated	Age, sex, education, and brain atrophy
Cherbuin et al 2015[37]	Cross-sectional	144<br>122	70.4 (1.4)<br>70.4 (1.4)	0<br>100	Occasional	150.3 (19.9)<br>148.9 (18.9)	82.4 (10)<br>79.5 (9.9)	NR	51.4<br>46.7	WM/GM regions	Semi-automated (VBM)	Age, sex, BMI, depression, and alcohol consumption.
DeCarli et al 1995[38]	Cross-sectional	51	52 (20)	49.0	Occasional	124 (14)	78 (9)	0	NR	WMLS	0.5 T/NR	Age and education
De Jong et al 2014[39]	Cross-sectional and Longitudinal	368	75.5 (5.3)	59.0	Occasional	143.1 (19.2)	74.5 (9.8)	54.0	59.0	MTL, BG	1.5 T/ Semi-automated	Age, sex, and ICV
Den Heijer et al 2005[2]	Cross-sectional and Longitudinal	511	73.4 (8)	49.1	Occasional	145.8 (20.3)	76.5 (11.6)	NR	38.9	HCV, Amygdala	1.5 T/ Manual	Age, sex and CVD factors
Den Heijer et al 2012[29]	Longitudinal	518	73.5 (7.9)	0	Occasional	145.9 (20.6)	76.7 (11.5)	53.0	39.0	lifte and right HCV	1.5 T/ Semi-automated	Age, sex
Dickie et al 2016[40]	Cross-sectional	681	72.7 (0.7)	47.0	Occasional	146 (18)	79 (9)	48.2	NR	WMLS	1.5 T/ Semi-automated	Sex, BMI, and CVD history

Study	Study Design	N	Age M (SD)	Sex (% female)	BP Methods	SBP M (SD)	DBP M (SD)	%HT	%AHT	Brain Region	Magnet / Segmentation	Covariables
Firbank et al 2007[5]	Cross-sectional	41	76 (4)	31.7	Occasional	133 (12)	73 (8)	0	0	WMLS, TBV	1.5 T/ Semi-automated	NR
Gattringer et al 2012[3]	Cross-sectional	287	66.6 (6.6)	49.8	Occasional	141.6 (21.8)	85.3 (9.7)	52.3	NR	WMLS, TBV, HCV	1.5 T/ Semi-automated	Age
Gianaros et al 2006[41]	Longitudinal	76<br>58	61.3 (5)<br>59.9 (5.1)	0<br>100	Occasional	132.4 (15.3)<br>128.8 (15)	79.3 (8.7)<br>76.6 (9.3)	38.0<br>22.0	NR	Regional GMV	1.5 T/ Semi-automated	Age and TBV
Glodzik et al 2014[24]	Cross-sectional	77	63.4 (9.4)	46.0	Occasional	NR	NR	39.0	0	HCV	1.5 T/ Semi-automated	Age, sex, education, ApoE status, and time to follow-up
Goldstein et al 2002[42]	Longitudinal	155	66.2 (6)	53.9	ABP	NR	NR	0	0	TBV, lateral Ventricles	1.5 T Manual/ TR blinded to information	
Goldstein et al 2005[18]	Longitudinal	121	66.2 (6)	57.0	Occasional	119.3 (13.8)	72.2 (8.9)	5.8	NR	TBV, WMLS, Insular subcortex	1.5 T Manual/ TR blinded to information	Age
Habes et al 2016[43]	Cross-sectional	2367	52.4 (13.7)	56.7	Occasional	127.3 (17.6)	NR	NR	32.7	WMLS	NR/ Semi-automated	Age, sex and education
Hajjar et al 2010[9]	Cross-sectional	43	68 (1)	56.0	Occasional	129 (2)	66 (1)	51.0	93.0	GMV/WM	3 T/ Semi-automated	Age, sex, race, BMI, and AHT medication
Haring et al 2019[23]	Longitudinal	558	78.3 (3.6)	100	Variability	122 (1)	73 (7)	48.0	NR	Regional GMV	3 T/ Semi-automated	Age, education, APOE4 allele
Hoogendam et al 2012[20]	Longitudinal	3962	60.1 (8.5)	54.4	Occasional	135.3 (19.5)	81.8 (10.7)	53.9	0	Cerebellar, Cerebral volume	1.5 T/ Semi-automated	Age, sex, and ICV
Ikram et al 2008[6]	Cross-sectional	490	73.4 (7.9)	50.8	Occasional	NR	NR	51.0	0	TBV, GMV, WMV	1.5 T/ Manual/ TR blinded to information	Age and sex.
Jeerakathil et al 2004[44]	Longitudinal	1814	53 (9.5)	53.0	Occasional	124.5 (18.2)	NR	18.3	0	WMLS	1 T/ Manual	Age and sex
Kern et al 2017[45]	Cross-sectional	64	72 (7)	67.2	Occasional	NR	NR	32.8	34.4	Regional GMV	1.5 T/ Semi-automated	Age, sex, education and general intellectual ability

Study	Study Design	N	Age M (SD)	Sex (% female)	BP Methods	SBP M (SD)	DBP M (SD)	%HT	%AHT	Brain Region	Magnet / Segmentation	Covariables
Kobuch et al 2020[46]	Cross-sectional	54	78.8 (1.5)	31.5	Occasional	NR	NR	NR	NR	Regional GMV	3 T/ Semi-automated (VBM)	Age, sex and ICV
Korf et al 2004[31]	Longitudinal	543	81.6 (5.0)	0	Occasional	NR	NR	25.8	NR	HCV	1.5 T/ Manual	Age, education, ApoE, smoking, alcohol, and dementia.
Lane et al 2019[13]	Cross-sectional and Longitudinal	441	69.0<br>60-64<br>53.0<br>43.0<br>36.0	49.0<br>49.0<br>49.0<br>49.0<br>49.0	Occasional	120.2 (13.7)<br>134.9 (16.9)<br>133.5 (19)<br>123.5 (13.7)<br>120.2 (13.7)	78.4 (9.5)<br>77 (9.4)<br>83.1 (11.8)<br>80.4 (9.3)<br>78.4 (9.5)	16.0<br>52.0<br>46.0<br>22.0<br>16.0	2.0<br>28.0<br>12.0<br>2.0<br>2.0	WMLS, TBV, HCV	3 T/ Semi-automated	Sex, APOE ε4 status, AHT medication, and BP at 69 years of age.
Launer et al 2015	Cross-sectional	680	50.3 (3.5)	52.2	Occasional	139.9 (1.5)	79.5 (0.9)	32.2	NR	WMLS, TBV	3 T/ Semi-automated	Age, sex, and race.
Mahinradet et al 2019[47]	Longitudinal itudinal	144	56 (4)	42.0	Occasional	107 (10)	65 (10)	48.6	30	WMLS	3 T/ Semi-automated	Age, sex, race, height, CVD factors, depression, and physical activity
McNeil et al 2018[16]	Cross-sectional	227	64.5 (0.8)	52.0	Occasional	139.9 (1.5)	79.5 (0.9)	NR	45.0	HCV	1.5 T/ Semi-automated	Age within this narrow age range sample.
Muller et al 2014[30]	Longitudinal	4057	50 (6)	59.0	Occasional	142 (13)	74 (6)	34.0	6.0	WMLS, TBV, GMV, WMV	1.5 T/ Semi-automated	Age, sex, education and CVD factors
Muller et al 2016[48]	Longitudinal	1348	50 (6)	58.0	Occasional	NR	NR	35.0	0	WMLS, TBV, GMV, WMV	1.5 T/ Semi-automated	Age, sex, education, and late-life CVD.
Nation et al 2016[21]	Longitudinal	549	59.6 (2.7)	53.2	Occasional	124 (16)	75 (9)	37.9	0	WMLS, TBV, HCV	1.5 T/ Semi-automated	Age, sex, and education
Paganini-Hill et al 2019[28]	Longitudinal	97	92.4 (0.3)	60	ABP	142 (1.5)	71 (1)	65.0	NA	WMLS	3 T/ Semi-automated	Age, sex, education, smoking and histories of CVD and cerebral vascular diseases
Pase et al 2016[22]	Cross-sectional	332	62.9 (10.2)	54.0	IDSBP	134 (19)	76 (10)	38.7	35.0	WMLS, TBV	1 T or 1.5 T/NR	Age, sex, and age

Study	Study Design	N	Age M (SD)	Sex (% female)	BP Methods	SBP M (SD)	DBP M (SD)	%HT	%AHT	Brain Region	Magnet / Segmentation	Covariables
Power et al 2016[49]	Cross-sectional and Longitudinal	1678	52.0	61.0	Occasional	130 (5.9)	66 (3.6)	23.0	72.0	TBV, HCV, brain lobes	3 T/ Semi-automated	Age, sex, race, education, ICV, BMI, DM, cholesterol, and smoking status
Sabayan et al 2013[4]	Longitudinal	553	74.9 (3.2)	43.6	Variability	156.1 (16.4)	85.1 (7.3)	63.1	NR	GMV, WM, HCV	1.5 T/ Semi-automated	Average BP and CVD factors
Schaare et al 2019[50]	Cross-sectional	423	27.7 (5.3)	41.8	Occasional	123.2 (12.2)	73.4 (8.5)	11.0	0	Regional G MV	3 T/ Semi-automated	Age. sex, and ICV
Scott et al 2015[32]	Cross-sectional	150	73.7 (6.3)	48.7	Occasional	136 (16)	75 (10)	44.0	NR	WMLS	3 T/NR	
Spartano et al 2016[51]	Longitudinal	1094	40 (9)	53.9	Exercise	166 (25.0)	74 (9)	28.3	17.7	TBV	1.5 T/NR	Age, sex, time between examination cycle and MRI, smoking, DM, APOE e4 genotype status, use of AHT medication; and serum homocysteine
Suzuki et al 2017[26]	Cross-sectional	1559	62.6 (7)	52.0	Occasional	125.6 (9.5)	74.5 (7.1)	0	0	TBV, GMV, WMV	3 T/ Semi-automated	Age, sex, education, BMI, and history of smoking, DM and CVD.
		1559	62.6 (6.8)	52.3		125.6 (9.5)	74.5 (7.1)	100	38.7			
Taki et al 2004[52]	Cross-sectional	769	47.4 (13.5)	53.8	Occasional	NR	NR	11.9	82.0	Regional G MV	0.5 T/ Semi-automated	
Taki et al 2013[53]	Longitudinal	381	51.2 (11.8)	59.0	Occasional	NR	NR	NR	NR	Regional G MV	0.5 T/ Semi-automated	Sex, ICV, SBP, and BMI
Trotman et al 2019[11]	Cross-sectional	40	19.1 (0.2)	100	Reactivity	122 (11.7)	77 (8.6)	NR	NR	HCV, Amygdala, Insula	3 T/ Semi-automated	Age, ICV, SES, and BMI
vanVelsen et al 2013[7]	Cross-sectional	1022	68.4 (7.3)	52.3	Occasional	144.5 (18.6)	80.3 (10.3)	47.4	0	Cortical thickness	1.5 T/ Semi-automated	Age and sex.
Verhaaren et al 2013[54]	Cross-sectional	665	61.6 (5)	52.0	Occasional	138 (19)	78 (10)	25.9	22.0	WMLS	1.5 T/ Semi-automated	Age, sex, and ICV, CVD factors

Study	Study Design	N	Age M (SD)	Sex (% female)	BP Methods	SBP M (SD)	DBP M (SD)	%HT	%AHT	Brain Region	Magnet / Segmentation	Covariables
Wardlaw et al 2014[35]	Cross-sectional	881	72.5 (0.7)	52.0	Occasional	120.2 (13.7)	78.4 (9.5)	49.0	NR	WMLS	1.5 T/ Semi-automated	Sex
White et al 2011[34]	Longitudinal	72	82.1 (3.9)	56.9	Occasional	122 (1.3)	73 (7)	70	64.0	WMLS	3 T/ Semi-automated	Age and LDL cholesterol levels,
Wiseman et al 2004[8]	Cross-sectional	154	77.2 (3.7)	78.6	Occasional	150 (16)	80 (9)	66.9	16.2	TBV, HCV	1.5 T/ Semi-automated	Age and ICV
Wolfson et al 2013[33]	Cross-sectional and Longitudinal	67	81.7 (3.9)	61.0	ASBP	138 (14)	69 (7)	NR	69.0	WMLS	3 T/ Semi-automated	Age, sex, and BMI or education
Yano et al 2017[10]	Longitudinal	547	25.6 (3.4)	53.9	Variability	123.2 (12.2)	73.4 (8.5)	51.8	21.2	TBV, GMV, WMV, HCV	3 T/ Semi-automated	Age, sex, ICV, antihypertensive medications, education, fasting glucose, smoking, and physical activity and BMI

M = mean; SD = standard deviation; SBP = Systolic Blood Pressure; DBP = Diastolic Blood Pressure; ASBP= ambulatory systolic blood pressure; ABP = ambulatory blood pressure; WMLS = White matter lesions; TBV = total brain volume; GMV= grey matter volume; WMV = white matter volume; HCV = Hippocampal volume; ICV = Intercranial volume; IPFC= ateral prefrontal cortex, pFWM = prefrontal white matter. CVD= Cardiovascular disease; Hypertension = HT; ATH = Antihypertensive; BMI = body mass index; DM = DM mellitus; WC = waist circumference, FBG = fasting blood glucose; APOE e4= Apolipoprotein E ; HDL-C= High-density lipoprotein cholesterol; LDL-C= low density lipoprotein-cholesterol, MetS= Metabolic syndrome; SES= socioeconomic status; T = tesla.

Table S3. Methodological quality of studies.

Studies	Total Rating	Methodological quality
Alkan et al 2019[36]	9.0/10.5	(85.7%) High
Allan et al 2015[17]	6.5/10.5	(61.9%) Moderate
Bender et al 2012[25]	6.5/10.5	(61.9%) Moderate
Brickman et al 2010[27]	7.5/10.5	(71.4%) High
Burns et al 2005[27]	2.0/10.5	(19%) Low
Cherbuin et al 2015[37]	8.0/10.5	(76.2%) High
DeCarli et al 1995[38]	0.5 /10.5	(76.2%) Low
De Jong et al 2014[39]	3.5 /10.5	(33.3%) Low
Den Heijeret al 2005[2]	2.5 /10.5	(23.8%) Low
Den Heijer et al 2012[29]	6.5 /10.5	(61.9%) Moderate
Dickie et al 2016[40]	5.0 /10.5	(47.6%) Moderate
Firbank et al 2007[5]	8.0 /10.5	(76.2%) High
Gattringer et al 2012[3]	1.5 /10.5	(14.3%) Low
Gianaros et al 2006[41]	8.0 /10.5	(76.2%) High
Glodzik et al 2014[24]	5.5 /10.5	(52.4%) Moderate
Goldstein et al 2002[42]	7.0 /10.5	(66.7%) Moderate
Goldstein et al 2005[18]	6.0 /10.5	(57.1%) Moderate
Habes et al 2016[43]	8.0 /10.5	(76.2%) High

Hajjar et al 2010[9]	8. /10.5 5	(81%) High
Haring et al 2019[23]	9.0 /10.5	(85.7%) High
Hoogendam et al 2012[20]	7.0 /10.5	(66.7%) Moderate
Ikram et al 2008[6]	5.5 /10.5	(52.4%) Moderate
Jeerakathil et al 2004[44]	3.0 /10.5	(28.6%) Low
Kern et al 2017[45]	9.0 /10.5	(85.7%) High
Kobuch et al 2020[46]	5.5 /10.5	(52.4%) Moderate
Korf et al 2004[31]	3.0 /10.5	(28.6%) Low
Lane et al 2019[13]	9.0 /10.5	(85.7%) High
Launer et al 2015[14]	6.5 /10.5	(61.9%) Moderate
Mahinradet al 2019[47]	10.0 /10.5	(95.2%) High
McNeil et al 2018[16]	6.5 /10.5	(61.9%) Moderate
Muller et al 2014[30]	6.0 /10.5	(57.1%) Moderate
Muller et al 2016[48]	5.0 /10.5	(47.6%) Moderate
Nation et al 2016[21]	8.0 /10.5	(76.2%) High
Paganini-Hill et al 2019[28]	4.0 /10.5	(38.1%) Low
Pase et al 2016[22]	8.0 /10.5	(76.2%) High
Power et al 2016[49]	5.0 /10.5	(47.6%) Moderate
Sabayan et al 2013[4]	5.0 /10.5	(47.6%) Moderate
Schaare et al 2019[50]	10.0 /10.5	(95.2%) High
Scott et al 2015[32]	2.0/10.5	(19%) Low
Spartano et al 2016[51]	4.0 /10.5	(38.1%) Low
Suzuki et al 2017[26]	9.0 /10.5	(85.7%) High
Taki et al 2004[52]	4.5 /10.5	(42.9%) Moderate
Taki et al 2013[53]	5.5 /10.5	(52.4%) Moderate
Trotman et al 2019[11]	2.5 /10.5	(23.8%) Low
Tsao et al 2016[19]	6.0 /10.5	(57.1%) Moderate
vanVelsen et al 2013[7]	5.0 /10.5	(47.6%) Moderate
Verhaaren et al 2013[54]	8.5 /10.5	(81.0%) High
Wardlaw et al 2014[35]	5.0 /10.5	(47.6%) Moderate
White et al 2011[34]	6.5 /10.5	(61.9%) Moderate
Wiseman et al 2004[8]	4.5 /10.5	(42.9%) Moderate
Wolfson et al 2013[33]	9.0 /10.5	(85.7%) High
Yano et al 2017[10]	9.5 /10.5	(90.5%) High

Meta-analysis results

White matter lesions volume (WMLS)

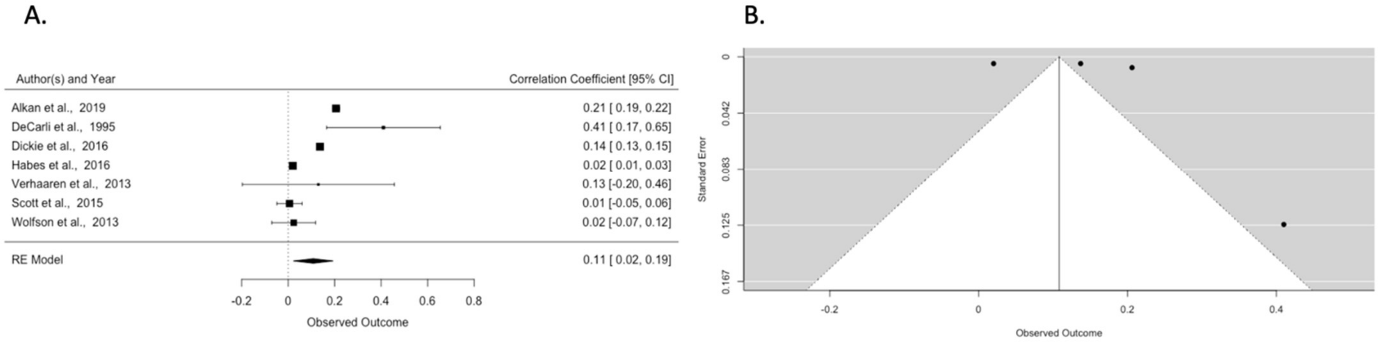

Figure S1. Association between SBP and White matter lesions from cross-sectional studies A. Forest plots; B. Sensitivity Analysis; trim and fill.

Random-Effects Model (k = 7; tau^2 estimator: REML)

logLik	deviance	AIC	BIC	AICc
3.6850	-7.3700	-3.3700	-3.7865	0.6300

tau^2 (estimated amount of total heterogeneity): 0.0102 (SE = 0.0073)
tau (square root of estimated tau^2 value): 0.1010
I^2 (total heterogeneity / total variability): 99.06%
H^2 (total variability / sampling variability): 106.59

Test for Heterogeneity:

Q(df = 6) = 506.2446, *p*-val < .0001

Model Results:

estimate	se	zval	pval	ci.lb	ci.ub	
0.1081	0.0435	2.4882	0.0128	0.0230	0.1933	*

Signif. codes: 0 '***' 0.001 '**' 0.01 '*' 0.05 '.' 0.1 ' ' 1

Sensitivity Analysis

Estimated number of missing studies on the left side: 0 (SE = 1.8715)
Random-Effects Model (k = 7; tau^2 estimator: REML)
tau^2 (estimated amount of total heterogeneity): 0.0102 (SE = 0.0073)
tau (square root of estimated tau^2 value): 0.1010
I^2 (total heterogeneity / total variability): 99.06%
H^2 (total variability / sampling variability): 106.59

Test for Heterogeneity:

Q(df = 6) = 506.2446, *p*-val < .0001

Model Results:

estimate	se	zval	pval	ci.lb	ci.ub	
0.1081	0.0435	2.4882	0.0128	0.0230	0.1933	*

Signif. codes: 0 '***' 0.001 '**' 0.01 '*' 0.05 '.' 0.1 ' ' 1

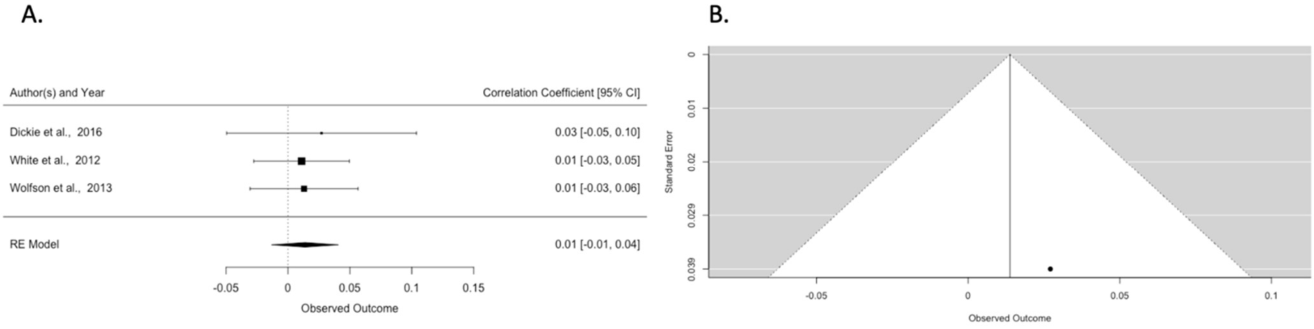

Figure S2. Association between SBP and White matter lesions from longitudinal studies. A. Forest plots; B. Sensitivity Anal and fill.

Random-Effects Model (k = 3; tau^2 estimator: REML)

logLik	deviance	AIC	BIC	AICc
5.3344	-10.6687	-6.6687	-9.2824	5.3313

tau^2 (estimated amount of total heterogeneity): 0 (SE = 0.0006)

tau (square root of estimated tau^2 value): 0
I^2 (total heterogeneity / total variability): 0.00%
H^2 (total variability / sampling variability): 1.00

Test for Heterogeneity:

Q(df = 2) = 0.1372, *p*-val = 0.9337

Model Results:

estimate	se	zval	pval	ci.lb	ci.ub
0.0138	0.0138	0.9984	0.3181	-0.0133	0.0408

Signif. codes: 0 '***' 0.001 '**' 0.01 '*' 0.05 '.' 0.1 ' ' 1

Sensitivity Analysis

Estimated number of missing studies on the left side: 0 (SE = 1.4967)
Random-Effects Model (k = 3; tau^2 estimator: REML)
tau^2 (estimated amount of total heterogeneity): 0 (SE = 0.0006)
tau (square root of estimated tau^2 value): 0
I^2 (total heterogeneity / total variability): 0.00%
H^2 (total variability / sampling variability): 1.00

Test for Heterogeneity:

Q(df = 2) = 0.1372, *p*-val = 0.9337

Model Results:

estimate	se	zval	pval	ci.lb	ci.ub
0.0138	0.0138	0.9984	0.3181	-0.0133	0.0408

Signif. codes: 0 '***' 0.001 '**' 0.01 '*' 0.05 '.' 0.1 ' ' 1

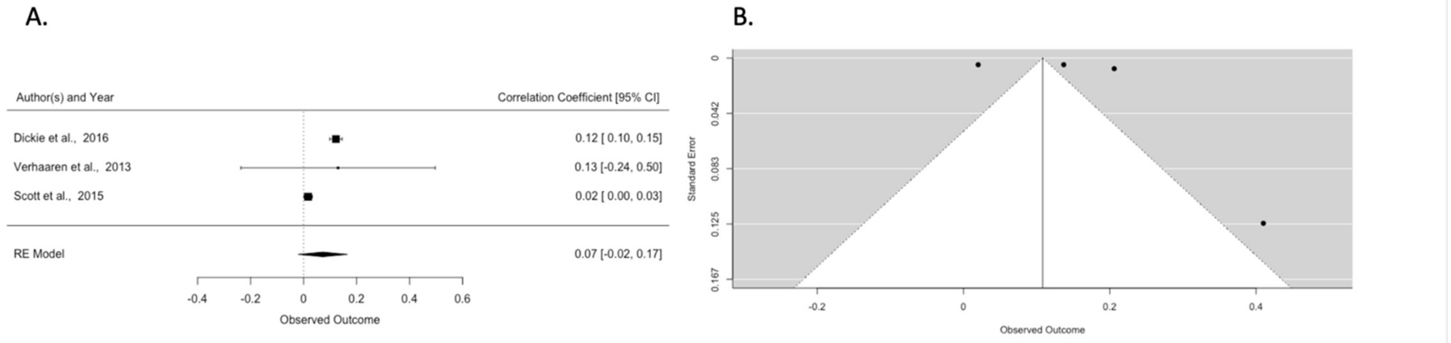

Figure S3. Association between DBP and White matter lesions from longitudinal studies. A. Forest plots; B. Sensitivity Analysis; trim and fill.

Random-Effects Model (k = 3; tau^2 estimator: REML)

logLik	deviance	AIC	BIC	AICc
1.9996	-3.9992	0.0008	-2.6129	12.0008

tau^2 (estimated amount of total heterogeneity): 0.0047 (SE = 0.0067)

tau (square root of estimated tau^2 value): 0.0683
I^2 (total heterogeneity / total variability): 95.69%
H^2 (total variability / sampling variability): 23.21

Test for Heterogeneity:

Q(df = 2) = 52.3723, p-val < .0001

Model Results:

estimate	se	zval	pval	ci.lb	ci.ub
0.0725	0.0475	1.5283	0.1264	-0.0205	0.1656

Signif. codes: 0 '***' 0.001 '**' 0.01 '*' 0.05 '.' 0.1 ' ' 1

Sensitivity Analysis

Estimated number of missing studies on the left side: 0 (SE = 1.8715)
Random-Effects Model (k = 7; tau^2 estimator: REML)
tau^2 (estimated amount of total heterogeneity): 0.0102 (SE = 0.0073)
tau (square root of estimated tau^2 value): 0.1010
I^2 (total heterogeneity / total variability): 99.06%
H^2 (total variability / sampling variability): 106.59

Test for Heterogeneity:

Q(df = 6) = 506.2446, *p*-val < .0001

Model Results:

estimate	se	zval	pval	ci.lb	ci.ub	
0.1081	0.0435	2.4882	0.0128	0.0230	0.1933	*

Signif. codes: 0 '***' 0.001 '**' 0.01 '*' 0.05 '.' 0.1 ' ' 1

Total brain volume (TBV)

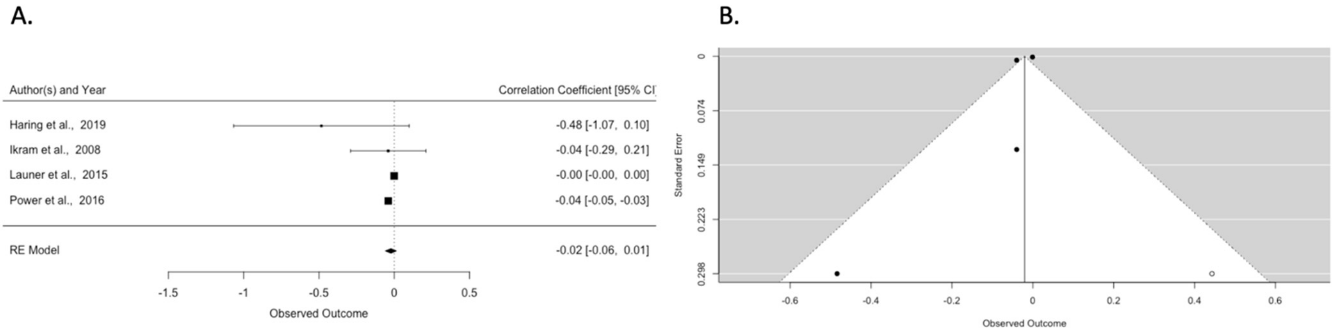

Figure S4. Association between SBP and total brain volume from cross-sectional studies. A. Forest plots; B. Sensitivity Analysis; trim and fill.

Random-Effects Model (k = 4; tau^2 estimator: REML)

logLik	deviance	AIC	BIC	AICc
2.6975	-5.3950	-1.3950	-3.1977	10.6050

tau^2 (estimated amount of total heterogeneity): 0.0007 (SE = 0.0010)
tau (square root of estimated tau^2 value): 0.0269
I^2 (total heterogeneity / total variability): 94.33%

H^2 (total variability / sampling variability): 17.63

Test for Heterogeneity:

Q(df = 3) = 55.4156, *p*-val < .0001

Model Results:

estimate	se	zval	pval	ci.lb	ci.ub
-0.0223	0.0190	-1.1762	0.2395	-0.0596	0.0149

Signif. codes: 0 '***' 0.001 '**' 0.01 '*' 0.05 '.' 0.1 ' ' 1

Sensitivity Analysis

Estimated number of missing studies on the right side: 1 (SE = 1.5779)
Random-Effects Model (k = 5; tau^2 estimator: REML)
tau^2 (estimated amount of total heterogeneity): 0.0007 (SE = 0.0010)
tau (square root of estimated tau^2 value): 0.0268
I^2 (total heterogeneity / total variability): 92.53%
H^2 (total variability / sampling variability): 13.38

Test for Heterogeneity:

Q(df = 4) = 57.6540, *p*-val < .0001

Model Results:

estimate	se	zval	pval	ci.lb	ci.ub
-0.0205	0.0189	-1.0834	0.2786	-0.0575	0.0166

Signif. codes: 0 '***' 0.001 '**' 0.01 '*' 0.05 '.' 0.1 ' ' 1

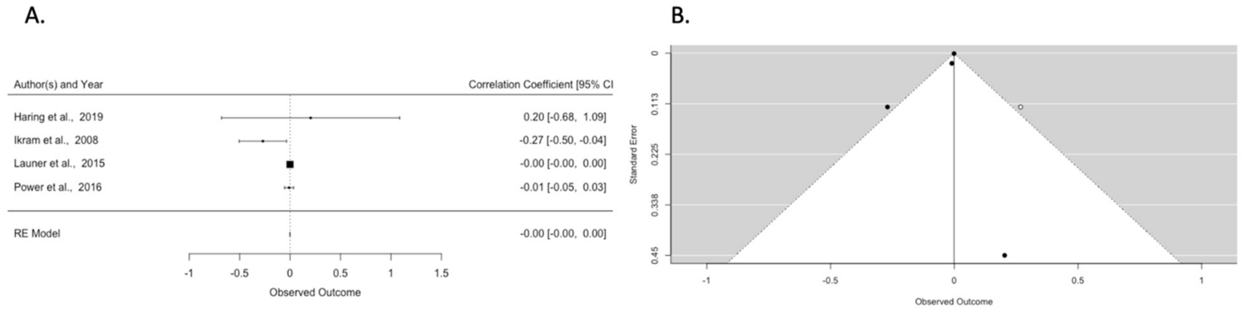

Figure S5. Association between DBP and total brain volume from cross-sectional studies. A. Forest plots; B. Sensitivity Analysis; trim and fill**.**

Random-Effects Model (k = 4; tau^2 estimator: REML)

logLik	deviance	AIC	BIC	AICc
1.9396	-3.8792	0.1208	-1.6820	12.1208

tau^2 (estimated amount of total heterogeneity): 0 (SE = 0.0004)
tau (square root of estimated tau^2 value): 0
I^2 (total heterogeneity / total variability): 0.00%
H^2 (total variability / sampling variability): 1.00

Test for Heterogeneity:

Q(df = 3) = 5.3948, *p*-val = 0.1451

Model Results:

estimate	se	zval	pval	ci.lb	ci.ub
-0.0010	0.0010	-1.0361	0.3002	-0.0030	0.0009

Signif. codes: 0 '***' 0.001 '**' 0.01 '*' 0.05 '.' 0.1 ' ' 1

Sensitivity Analysis

Estimated number of missing studies on the right side: 1 (SE = 1.6103)
Random-Effects Model (k = 5; tau^2 estimator: REML)
tau^2 (estimated amount of total heterogeneity): 0 (SE = 0.0004)
tau (square root of estimated tau^2 value): 0
I^2 (total heterogeneity / total variability): 0.00%
H^2 (total variability / sampling variability): 1.00

Test for Heterogeneity:

Q(df = 4) = 10.4280, *p*-val = 0.0338

Model Results:

estimate	se	zval	pval	ci.lb	ci.ub
-0.0010	0.0010	-1.0174	0.3090	-0.0030	0.0009

Signif. codes: 0 '***' 0.001 '**' 0.01 '*' 0.05 '.' 0.1 ' ' 1

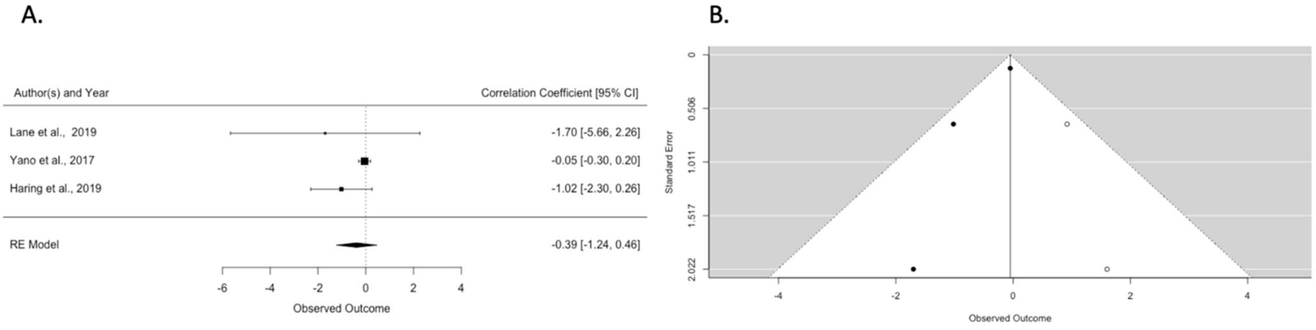

Figure S6. Association between SBP variability and total brain volume from longitudinal studies. A. Forest plots; B. Sensitivity Analysis; trim and fill.

Random-Effects Model (k = 3; tau^2 estimator: REML)

logLik	deviance	AIC	BIC	AICc
-2.7216	5.4432	9.4432	6.8295	21.4432

tau^2 (estimated amount of total heterogeneity): 0.2601 (SE = 0.6657)
tau (square root of estimated tau^2 value): 0.5100
I^2 (total heterogeneity / total variability): 39.31%
H^2 (total variability / sampling variability): 1.65

Test for Heterogeneity:

Q(df = 2) = 2.7519, *p*-val = 0.2526

Model Results:

estimate	se	zval	pval	ci.lb	ci.ub
-0.3862	0.4342	-0.8895	0.3738	-1.2371	0.4648

Signif. codes: 0 '***' 0.001 '**' 0.01 '*' 0.05 '.' 0.1 ' ' 1

Sensitivity Analysis

Estimated number of missing studies on the right side: 2 (SE = 1.4881)
Random-Effects Model (k = 5; tau^2 estimator: REML)
tau^2 (estimated amount of total heterogeneity): 0.1990 (SE = 0.4248)
tau (square root of estimated tau^2 value): 0.4461
I^2 (total heterogeneity / total variability): 32.73%
H^2 (total variability / sampling variability): 1.49

Test for Heterogeneity:

Q(df = 4) = 5.7247, *p*-val = 0.2207

Model Results:

estimate	se	zval	pval	ci.lb	ci.ub
-0.0490	0.3470	-0.1412	0.8877	-0.7290	0.6310

Signif. codes: 0 '***' 0.001 '**' 0.01 '*' 0.05 '.' 0.1 ' ' 1

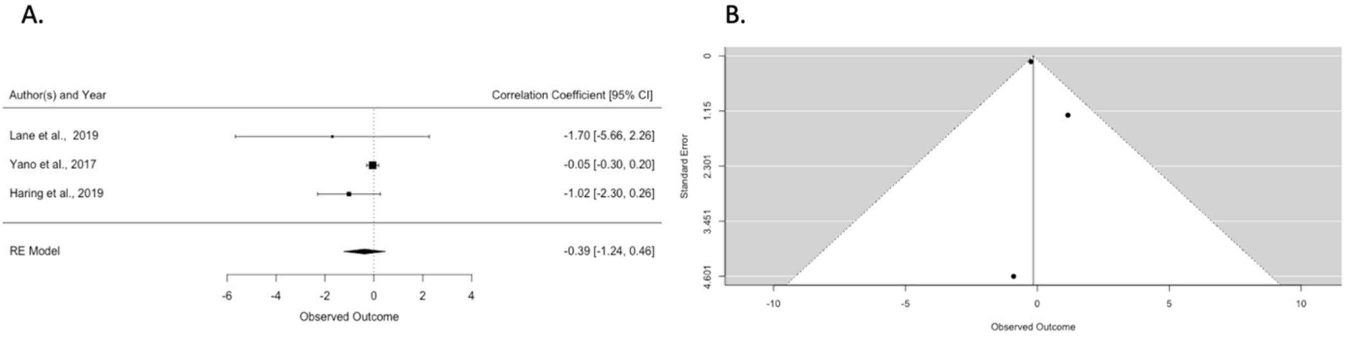

Figure S7. Association between DBP variability and total brain volume from longitudinal studies. A. Forest plots; B. Sensitivity Analysis; trim and fill.

Random-Effects Model (k = 3; tau^2 estimator: REML)

logLik	deviance	AIC	BIC	AICc
-3.6756	7.3513	11.3513	8.7376	23.3513

tau^2 (estimated amount of total heterogeneity): 0.1079 (SE = 1.2247)
tau (square root of estimated tau^2 value): 0.3285
I^2 (total heterogeneity / total variability): 6.96%
H^2 (total variability / sampling variability): 1.07

Test for Heterogeneity:

Q(df = 2) = 1.2944, p-val = 0.5235

Model Results:

estimate	se	zval	pval	ci.lb	ci.ub
-0.1526	0.3365	-0.4536	0.6501	-0.8121	0.5069

Signif. codes: 0 '***' 0.001 '**' 0.01 '*' 0.05 '.' 0.1 ' ' 1

Sensitivity Analysis

Estimated number of missing studies on the left side: 0 (SE = 1.4967)
Random-Effects Model (k = 3; tau^2 estimator: REML)
tau^2 (estimated amount of total heterogeneity): 0.1079 (SE = 1.2247)
tau (square root of estimated tau^2 value): 0.3285
I^2 (total heterogeneity / total variability): 6.96%
H^2 (total variability / sampling variability): 1.07

Test for Heterogeneity:

Q(df = 2) = 1.2944, p-val = 0.5235

Model Results

estimate	se	zval	pval	ci.lb	ci.ub
-0.1526	0.3365	-0.4536	0.6501	-0.8121	0.5069

Signif. codes: 0 '***' 0.001 '**' 0.01 '*' 0.05 '.' 0.1 ' ' 1

Hippocampal volume (HCV)

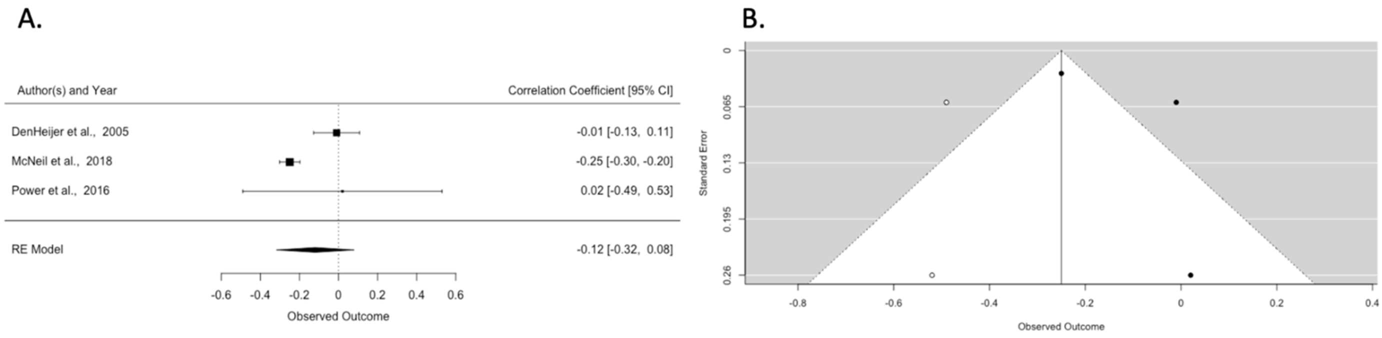

Figure S8. Association between DBP and hippocampal volume from cross-sectional studies. A. Forest plots; B. Sensitivity Analysis; trim and fill.

Random-Effects Model (k = 3; tau^2 estimator: REML)

logLik	deviance	AIC	BIC	AICc
0.6525	-1.3049	2.6951	0.0813	14.6951

tau^2 (estimated amount of total heterogeneity): 0.0211 (SE = 0.0310)
tau (square root of estimated tau^2 value): 0.1453
I^2 (total heterogeneity / total variability): 83.80%
H^2 (total variability / sampling variability): 6.17

Test for Heterogeneity:

Q(df = 2) = 14.1697, p-val = 0.0008

Model Results:

estimate	se	zval	pval	ci.lb	ci.ub
-0.1193	0.1012	-1.1787	0.2385	-0.3177	0.0791

Signif. codes: 0 '***' 0.001 '**' 0.01 '*' 0.05 '.' 0.1 ' ' 1

Sensitivity Analysis

Estimated number of missing studies on the left side: 2 (SE = 1.4881)
Random-Effects Model (k = 5; tau^2 estimator: REML)
tau^2 (estimated amount of total heterogeneity): 0.0432 (SE = 0.0413)
tau (square root of estimated tau^2 value): 0.2078
I^2 (total heterogeneity / total variability): 90.88%
H^2 (total variability / sampling variability): 10.97

Test for Heterogeneity:

Q(df = 4) = 34.1568, *p*-val < .0001

Model Results:

estimate	se	zval	pval	ci.lb	ci.ub	
-0.2500	0.1094	-2.2854	0.0223	-0.4644	-0.0356	*

Signif. codes: 0 '***' 0.001 '**' 0.01 '*' 0.05 '.' 0.1 ' ' 1

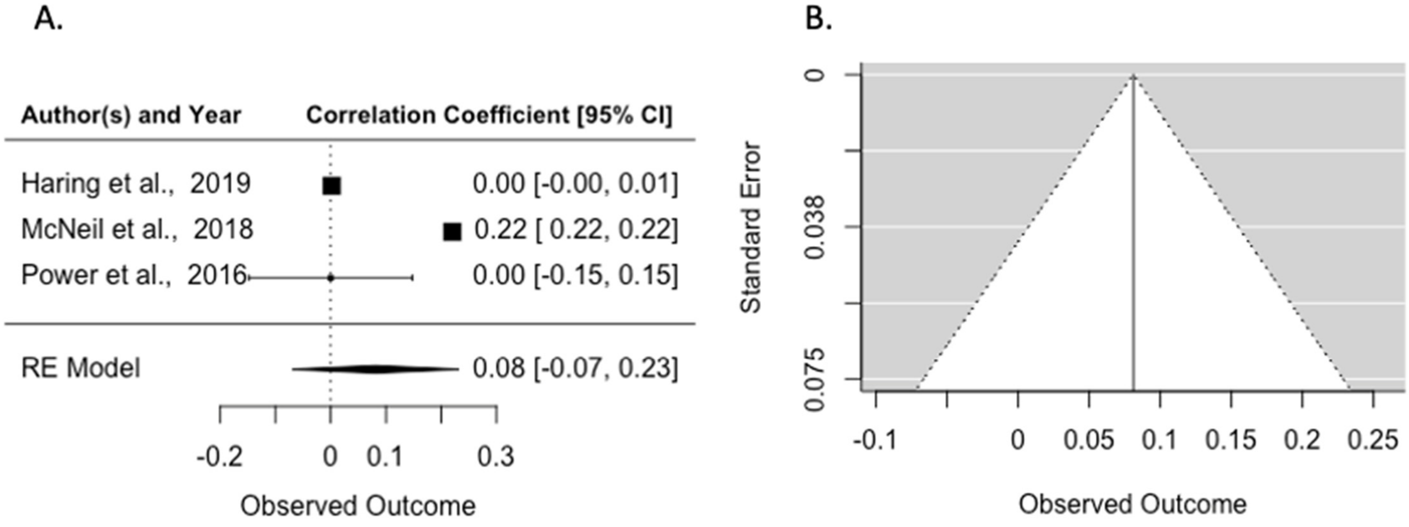

Figure S9. Association between DBP and hippocampal volume from cross-sectional studies. A. Forest plots; B. Sensitivity Analysis: trim and fill.

Random-Effects Model (k = 3; tau^2 estimator: REML)

logLik	deviance	AIC	BIC	AICc
1.2400	-2.4800	1.5200	-1.0937	13.5200

tau^2 (estimated amount of total heterogeneity): 0.0161 (SE = 0.0177)
tau (square root of estimated tau^2 value): 0.1270

Model Results:

estimate	se	zval	pval	ci.lb	ci.ub
0.0810	0.0767	1.0554	0.2912	-0.0694	0.2313

Signif. codes: 0 '***' 0.001 '**' 0.01 '*' 0.05 '.' 0.1 ' ' 1

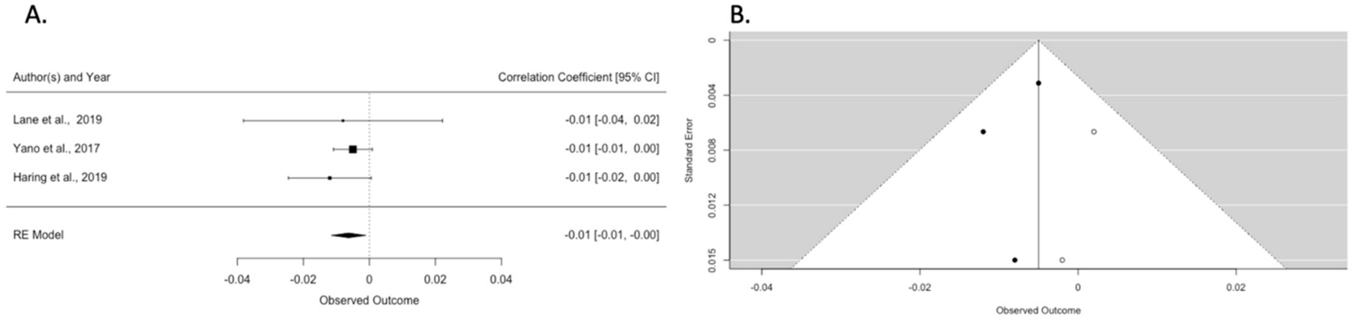

Figure S10. Association between SBP variability and hippocampal volume from longitudinal studies. A. Forest plots; B. Sensitivity Analysis; trim and fill.

Random-Effects Model (k = 3; tau^2 estimator: REML)

logLik	deviance	AIC	BIC	AICc
7.3265	-14.6529	-10.6529	-13.2666	1.3471

tau^2 (estimated amount of total heterogeneity): 0 (SE = 0.0000)
tau (square root of estimated tau^2 value): 0
I^2 (total heterogeneity / total variability): 0.00%
H^2 (total variability / sampling variability): 1.00

Test for Heterogeneity:

Q(df = 2) = 0.9932, *p*-val = 0.6086

Model Results:

estimate	se	zval	pval	ci.lb	ci.ub	
-0.0063	0.0027	-2.3603	0.0183	-0.0116	-0.0011	*

Signif. codes: 0 '***' 0.001 '**' 0.01 '*' 0.05 '.' 0.1 ' ' 1

Sensitivity Analysis

Estimated number of missing studies on the right side: 2 (SE = 1.4881)
Random-Effects Model (k = 5; tau^2 estimator: REML)
tau^2 (estimated amount of total heterogeneity): 0 (SE = 0.0000)
tau (square root of estimated tau^2 value): 0
I^2 (total heterogeneity / total variability): 0.00%
H^2 (total variability / sampling variability): 1.00

Test for Heterogeneity:

Q(df = 4) = 2.4687, *p*-val = 0.6502

Model Results:

estimate	se	zval	pval	ci.lb	ci.ub	
-0.0050	0.0024	-2.0518	0.0402	-0.0098	-0.0002	*

Signif. codes: 0 '***' 0.001 '**' 0.01 '*' 0.05 '.' 0.1 ' ' 1

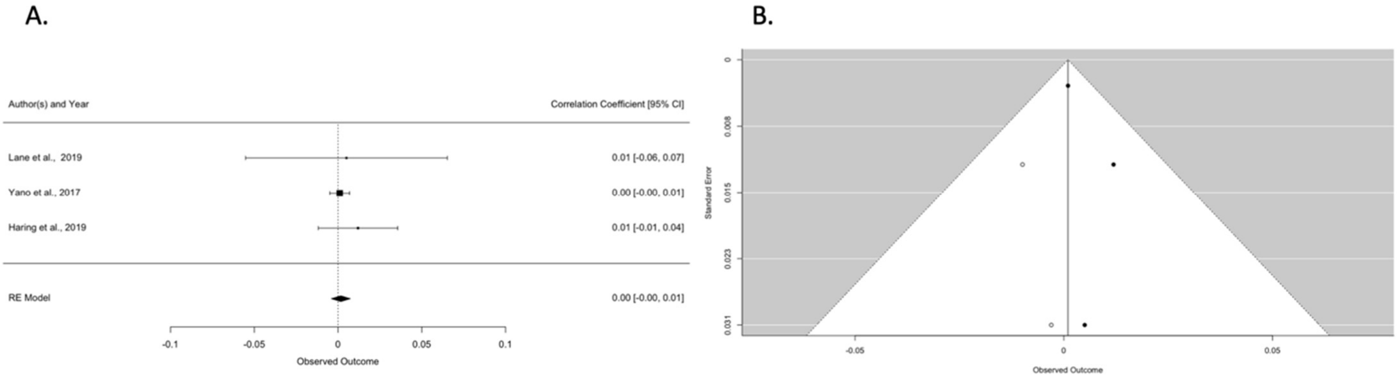

Figure S11. Association between DBP variability and hippocampal volume from longitudinal studies. A. Forest plots; B. Sensitivity Analysis; trim and fill.

Random-Effects Model (k = 3; tau^2 estimator: REML)

logLik	deviance	AIC	BIC	AICc
6.1862	-12.3723	-8.3723	-10.9860	3.6277

tau^2 (estimated amount of total heterogeneity): 0 (SE = 0.0001)
tau (square root of estimated tau^2 value): 0
I^2 (total heterogeneity / total variability): 0.00%
H^2 (total variability / sampling variability): 1.00

Test for Heterogeneity:

Q(df = 2) = 0.7764, *p*-val = 0.6783

Model Results:

estimate	se	zval	pval	ci.lb	ci.ub
0.0017	0.0029	0.5731	0.5666	-0.0040	0.0073

Signif. codes: 0 '***' 0.001 '**' 0.01 '*' 0.05 '.' 0.1 ' ' 1
Estimated number of missing studies on the left side: 2 (SE = 1.4881)
Random-Effects Model (k = 5; tau^2 estimator: REML)
tau^2 (estimated amount of total heterogeneity): 0 (SE = 0.0001)
tau (square root of estimated tau^2 value): 0
I^2 (total heterogeneity / total variability): 0.00%
H^2 (total variability / sampling variability): 1.00

Test for Heterogeneity:

Q(df = 4) = 1.6569, *p*-val = 0.7985

Model Results:

estimate	se	zval	pval	ci.lb	ci.ub
0.0010	0.0028	0.3562	0.7217	-0.0045	0.0065

Signif. codes: 0 '***' 0.001 '**' 0.01 '*' 0.05 '.' 0.1 ' '

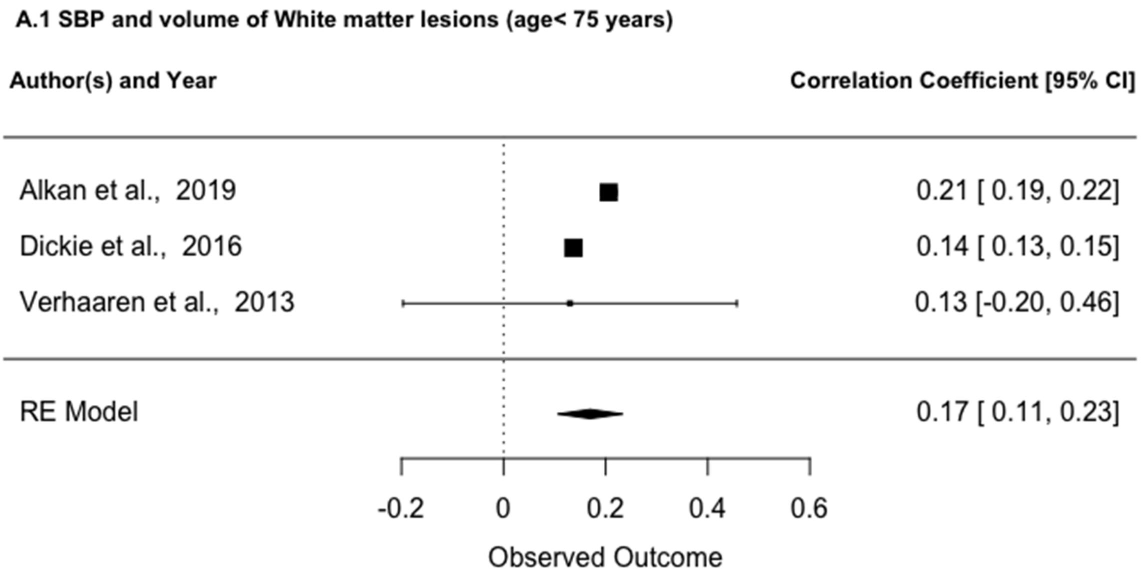

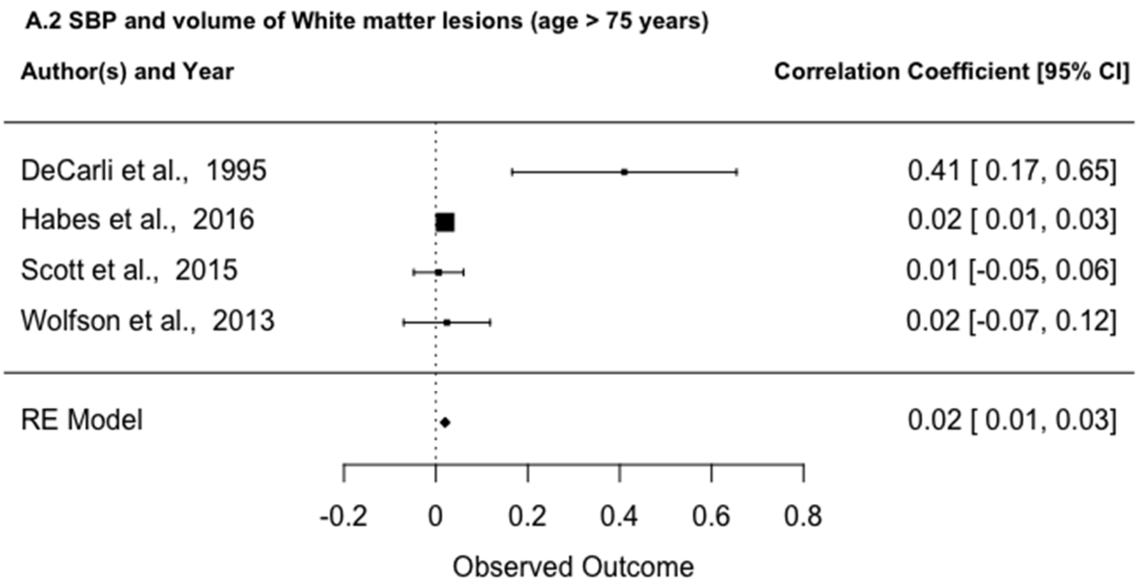

Figure S12. The Forest plots show the association between SBP and white matter lesions in elderly below or above ~75 years. Given the small number of studies these results should be interpreted with caution. However, the pattern of results appears to indicate that effects are consistent below in younger individuals (mean weighted age ~72 years). In contrast, while still significant in older individuals (mean weighted age 80.6 years) the effect appears much reduced in this age group.

References

1. Burns, J.M.; Church, J.A.; Johnson, D.K.; Xiong, C.; Marcus, D.; Fotenos, A.F.; Snyder, A.Z.; Morris, J.C.; Buckner, R.L. White matter lesions are prevalent but differentially related with cognition in aging and early Alzheimer

disease. *Arch. Neurol.* **2005**, *62*, 1870–1876, doi:10.1001/archneur.62.12.1870.

2. den Heijer, T.; Launer, L.J.; Prins, N.D.; van Dijk, E.J.; Vermeer, S.E.; Hofman, A.; Koudstaal, P.J.; Breteler, M.M. Association between blood pressure, white matter lesions, and atrophy of the medial temporal lobe. *Neurology* **2005**, *64*, 263–267, doi:10.1212/01.Wnl.0000149641.55751.2e.
3. Gattringer, T.; Enzinger, C.; Ropele, S.; Gorani, F.; Petrovic, K.E.; Schmidt, R.; Fazekas, F. Vascular risk factors, white matter hyperintensities and hippocampal volume in normal elderly individuals. *Dement. Geriatr. Cogn. Disord.* **2012**, *33*, 29–34, doi:10.1159/000336052.
4. Sabayan, B.; Wijsman, L.W.; Foster-Dingley, J.C.; Stott, D.J.; Ford, I.; Buckley, B.M.; Sattar, N.; Jukema, J.W.; van Osch, M.J.; van der Grond, J. et al. Association of visit-to-visit variability in blood pressure with cognitive function in old age:prospective cohort study. *BMJ(Clinical Res. ed.)* **2013**, *347*, f4600.
5. Firbank, M.J.; Wiseman, R.M.; Burton, E.J.; Saxby, B.K.; O'Brien, J.T.; Ford, G.A. Brain atrophy and white matter hyperintensity change in older adults and relationship to blood pressure. *J. Neurol.* **2007**, doi:10.1007/s00415-006-0238-4.
6. Ikram, M.A.; Vrooman, H.A.; Vernooij, M.W.; van der Lijn, F.; Hofman, A.; van der Lugt, A.; Niessen, W.J.; Breteler, M.M.B. Brain tissue volumes in the general elderly population. The Rotterdam Scan Study. *Neurobiol. Aging* **2008**, *29*, 882–890, doi:10.1016/j.neurobiolaging.2006.12.012.
7. van Velsen, E.F.S.; Vernooij, M.W.; Vrooman, H.A.; van der Lugt, A.; Breteler, M.M.B.; Hofman, A.; Niessen, W.J.; Ikram, M.A. Brain cortical thickness in the general elderly population: The Rotterdam Scan Study. *Neurosci. Lett.* **2013**, doi:10.1016/j.neulet.2013.06.063.
8. Wiseman, R.M.; Saxby, B.K.; Burton, E.J.; Barber, R.; Ford, G.A.; Brien, J.T.O. Hippocampal atrophy , whole brain volume , and white matter lesions in older hypertensive subjects. **2004**.
9. Hajjar, I.; Zhao, P.; Alsop, D.; Abduljalil, A.; Selim, M.; Novak, P.; Novak, V. Association of blood pressure elevation and nocturnal dipping with brain atrophy Perfusion and Functional Measures in Stroke and Nonstroke Individuals. *Am. J. Hypertens.* **2010**, *23*, 17–23, doi:10.1038/ajh.2009.187.
10. Yano, Y.; Reis, J.P.; Levine, D.A.; Bryan, R.N.; Viera, A.J.; Shimbo, D.; Tedla, Y.G.; Allen, N.B.; Schreiner, P.J.; Bancks, M.P.; et al. Visit-to-Visit Blood Pressure Variability in Young Adulthood and Hippocampal Volume and Integrity at Middle Age. *Hypertens. (Dallas, Tex. 1979)* **2017**, *70*, 1091–1098, doi:10.1161/hypertensionaha.117.10144.
11. Trotman, G.P.; Williams, S.E.; Ginty, A.T.; Gianaros, P.J. Increased stressor - evoked cardiovascular reactivity is associated with reduced amygdala and hippocampus volume. **2019**, doi:10.1111/psyp.13277.
12. Schaare, H.L.; Kharabian Masouleh, S.; Beyer, F.; Kumral, D.; Uhlig, M.; Reinelt, J.D.; Reiter, A.M.F.; Lampe, L.; Babayan, A.; Al, E. Association of peripheral blood pressure with gray matter volume in 19- to 40-year-old adults. *Neurology* **2019**, doi:10.1212/WNL.0000000000006947.
13. Lane, C.A.; Barnes, J.; Nicholas, J.M.; Sudre, C.H.; Cash, D.M.; Parker, T.D.; Malone, I.B.; Lu, K.; James, S.-N.;

Keshavan, et al. Associations between blood pressure across adulthood and late-life brain structure and pathology in the neuroscience substudy of the 1946 British birth cohort (Insight 46): an epidemiological study. *Lancet. Neurol.* **2019**, *18*, 942–952, doi:10.1016/S1474-4422(19)30228-5.

14. Launer, L.J.; Lewis, C.E.; Schreiner, P.J.; Sidney, S.; Battapady, H.; Jacobs, D.R.; Lim, K.O.; D'Esposito, M.; Zhang, Q.; Reis, J.; et al. Vascular factors and multiple measures of early brain health: CARDIA Brain MRI Study. *PLoS One* **2015**, *10*, e0122138, doi:10.1371/journal.pone.0122138.
15. Swan, G.E.; DeCarli, C.; Miller, B.L.; Reed, T.; Wolf, P.A.; Jack, L.M.; Carmelli, D. Association of midlife blood pressure to late-life cognitive decline and brain morphology. *Neurology* **1998**, doi:10.1212/WNL.51.4.986.
16. McNeil, C.J.; Myint, P.K.; Sandu, A.L.; Potter, J.F.; Staff, R.; Whalley, L.J.; Murray, A.D. Increased diastolic blood pressure is associated with MRI biomarkers of dementia-related brain pathology in normative ageing. *Age Ageing* **2018**, *47*, 95–100, doi:10.1093/ageing/afx102.
17. Allan, C.L.; Zsoldos, E.; Filippini, N.; Sexton, C.E.; Topiwala, A.; Valkanova, V.; Singh-Manoux, A.; Tabák, A.G.; Shipley, M.J.; Mackay, C.; et al. Lifetime hypertension as a predictor of brain structure in older adults: Cohort study with a 28-year follow-up. *Br. J. Psychiatry* **2015**, *206*, 308–315, doi:10.1192/bjp.bp.114.153536.
18. Goldstein, I.B.; Bartzokis, G.; Guthrie, D.; Shapiro, D. Ambulatory blood pressure and the brain: A 5-year follow-up. *Neurology* **2005**, *64*, 1846–1852, doi:10.1212/01.WNL.0000164712.24389.BB.
19. Tsao, C.W.; Himali, J.J.; Beiser, A.S.; Larson, M.G.; Decarli, C.; Mitchell, G.F. Association of arterial stiffness with progression of subclinical brain and cognitive disease. **2016**, 619–626.
20. Hoogendam, Y.Y.; van der Geest, J.N.; van der Lijn, F.; van der Lugt, A.; Niessen, W.J.; Krestin, G.P.; Hofman, A.; Vernooij, M.W.; Breteler, M.M.; Ikram, M.A. Determinants of cerebellar and cerebral volume in the general elderly population. *Neurobiol. Aging* **2012**, *33*, 2774–2781, doi:10.1016/j.neurobiolaging.2012.02.012.
21. Nation, D.A.; Preis, S.R.; Beiser, A.; Bangen, K.J.; Delano-Wood, L.; Lamar, M.; Libon, D.J.; Seshadri, S.; Wolf, P.A.; Au, R. Pulse Pressure Is Associated With Early Brain Atrophy and Cognitive Decline: Modifying Effects of APOE-epsilon4. *Alzheimer Dis. Assoc. Disord.* **2016**, *30*, 210–215, doi:10.1097/wad.0000000000000127.
22. Pase, M.P.; Beiser, A.; Aparicio, H.; DeCarli, C.; Vasan, R.S.; Murabito, J.; Seshadri, S. Interarm differences in systolic blood pressure and the risk of dementia and subclinical brain injury. *Alzheimers. Dement.* **2016**, *12*, 438–445, doi:10.1016/j.jalz.2015.09.006.
23. Haring, B.; Liu, J.; Salmoirago-Blotcher, E.; Hayden, K.M.; Sarto, G.; Roussouw, J.; Kuller, L.H.; Rapp, S.R.; Wassertheil-Smoller, S. Blood pressure variability and brain morphology in elderly women without cardiovascular disease. *Neurology* **2019**, *92*, E1284–E1297, doi:10.1212/WNL.0000000000007135.
24. Glodzik, L.; Rusinek, H.; Pirraglia, E.; McHugh, P.; Tsui, W.; Williams, S.; Cummings, M.; Li, Y.; Rich, K.; Randall, C.; et al. Blood pressure decrease correlates with tau pathology and memory decline in hypertensive elderly. *Neurobiol. Aging* **2014**, *35*, 64–71, doi:10.1016/j.neurobiolaging.2013.06.011.
25. Bender, A.R.; Raz, N. Age-related differences in memory and executive functions in healthy APOE varepsilon4

carriers: the contribution of individual differences in prefrontal volumes and systolic blood pressure. *Neuropsychologia* **2012**, *50*, 704–714, doi:10.1016/j.neuropsychologia.2011.12.025.

26. Suzuki, H.; Gao, H.; Bai, W.; Evangelou, E.; Glocker, B.; O'Regan, D.P.; Elliott, P.; Matthews, P.M. Abnormal brain white matter microstructure is associated with both pre-hypertension and hypertension. *PLoS One* **2017**, *12*.
27. Brickman, A.M.; Reitz, C.; Luchsinger, J.A.; Manly, J.J.; Schupf, N.; Muraskin, J.; DeCarli, C.; Brown, T.R.; Mayeux, R. Long-term blood pressure fluctuation and cerebrovascular disease in an elderly cohort. *Arch. Neurol.* **2010**, *67*, 564–569, doi:10.1001/archneurol.2010.70.
28. Paganini-Hill, A.; Bryant, N.; Corrada, M.M.; Greenia, D.E.; Fletcher, E.; Singh, B.; Floriolli, D.; Kawas, C.H.; Fisher, M.J. Blood Pressure Circadian Variation, Cognition and Brain Imaging in 90+ Year-Olds. *Front. Aging Neurosci.* **2019**, *11*, 54, doi:10.3389/fnagi.2019.00054.
29. Heijer, T. Den; Lijn, F. Van Der; Ikram, A.; Koudstaal, P.J.; Lugt, A. Van Der; Krestin, G.P.; Vrooman, H.A.; Hofman, A.; Niessen, W.J.; Breteler, M.M.B. Vascular risk factors , apolipoprotein E , and hippocampal decline on magnetic resonance imaging over a 10-year follow-up. *Alzheimer's Dement.* **2012**, *8*, 417–425, doi:10.1016/j.jalz.2011.07.005.
30. Muller, M.; Sigurdsson, S.; Kjartansson, O.; Aspelund, T.; Lopez, O.L.; Jonnson, P. V.; Harris, T.B.; Van Buchem, M.; Gudnason, V.; Launer, L.J. Joint effect of mid- and late-life blood pressure on the brain: The AGES-Reykjavik Study. *Neurology* **2014**, *82*, 2187–2195, doi:10.1212/WNL.0000000000000517.
31. Korf, E.S.; White, L.R.; Scheltens, P.; Launer, L.J. Midlife blood pressure and the risk of hippocampal atrophy-The Honolulu Asia Aging Study. *Hypertens. (Dallas, Tex. 1979)* **2004**, *44*, 29–34, doi:10.1161/01.HYP.0000132475.32317.bb.
32. Scott, J.A.; Braskie, M.N.; Tosun, D.; Thompson, P.M.; Weiner, M.; DeCarli, C.; Carmichael, O.T. Cerebral amyloid and hypertension are independently associated with white matter lesions in elderly. *Front. Aging Neurosci.* **2015**, *7*, doi:10.3389/fnagi.2015.00221.
33. Wolfson, L.; Wakefield, D.B.; Moscufo, N.; Kaplan, R.F.; Hall, C.B.; Schmidt, J.A.; Guttmann, C.R.G.; White, W.B. Rapid buildup of brain white matter hyperintensities over 4 years linked to ambulatory blood pressure, mobility, cognition, and depression in old persons. *Journals Gerontol. - Ser. A Biol. Sci. Med. Sci.* **2013**, *68*, 1387–1394, doi:10.1093/gerona/glt072.
34. White, W.B.; Wolfson, L.; Wakefield, D.B.; Hall, C.B.; Campbell, P.; Moscufo, N.; Schmidt, J.; Kaplan, R.F.; Pearlson, G.; Guttmann, C.R. Average daily blood pressure, not office blood pressure, is associated with progression of cerebrovascular disease and cognitive decline in older people. *Circulation* **2011**, *124*, 2312–2319, doi:10.1161/circulationaha.111.037036.
35. Wardlaw, J.M.; Allerhand, M.; Doubal, F.N.; Hernandez, M.V.; Morris, Z.; Gow, A.J.; Bastin, M.; Starr, J.M.; Dennis, M.S.; Deary, I.J. Vascular risk factors , large-artery atheroma , and brain white matter hyperintensities. **2014**,

1331–1338.

36. Alkan, E.; Taporoski, T.P.; Sterr, A.; von Schantz, M.; Vallada, H.; Krieger, J.E.; Pereira, A.C.; Alvim, R.; Horimoto, A.R.V.R.; Pompéia, S.; et al. Metabolic syndrome alters relationships between cardiometabolic variables, cognition and white matter hyperintensity load. *Sci. Rep.* **2019**, *9*, 1–9, doi:10.1038/s41598-019-40630-6.
37. Cherbuin, N.; Mortby, M.E.; Janke, A.L.; Sachdev, P.S.; Abhayaratna, W.P.; Anstey, K.J. Blood Pressure, Brain Structure, and Cognition: Opposite Associations in Men and Women. *Am. J. Hypertens.* **2015**, *28*, 225–231, doi:10.1093/ajh/hpu120.
38. DeCarli, C.; Murphy, D.G.; Tranh, M.; Grady, C.L.; Haxby, J. V; Gillette, J.A.; Salerno, J.A.; Gonzales-Aviles, A.; Horwitz, B.; Rapoport, S.I.; et al. The effect of white matter hyperintensity volume on brain structure, cognitive performance, and cerebral metabolism of glucose in 51 healthy adults. *Neurology* **1995**, *45*, 2077–2084.
39. de Jong, L.W.; Forsberg, L.E.; Vidal, J.S.; Sigurdsson, S.; Zijdenbos, A.P.; Garcia, M.; Eiriksdottir, G.; Gudnason, V.; van Buchem, M.A.; Launer, L.J. Different susceptibility of medial temporal lobe and basal ganglia atrophy rates to vascular risk factors. *Neurobiol. Aging* **2014**, *35*, 72–78, doi:10.1016/j.neurobiolaging.2013.07.009.
40. Dickie, D.A.; Ritchie, S.J.; Cox, S.R.; Sakka, E.; Royle, N.A.; Aribisala, B.S.; Valdés Hernández, M. del C.; Maniega, S.M.; Pattie, A.; Corley, J.; et al. Vascular risk factors and progression of white matter hyperintensities in the Lothian Birth Cohort 1936. *Neurobiol. Aging* **2016**, *42*, 116–123, doi:10.1016/j.neurobiolaging.2016.03.011.
41. Gianaros, P.J.; Greer, P.J.; Ryan, C.M.; Jennings, J.R. Higher blood pressure predicts lower regional grey matter volume: Consequences on short-term information processing. *Neuroimage* **2006**, *31*, 754–765, doi:10.1016/j.neuroimage.2006.01.003.
42. Goldstein, I.B.; Bartzokis, G.; Guthrie, D.; Shapiro, D. Ambulatory blood pressure and brain atrophy in the healthy elderly. *Neurology* **2002**, *59*, 713–719.
43. Habes, M.; Erus, G.; Toledo, J.B.; Zhang, T.; Bryan, N.; Launer, L.J.; Rosseel, Y.; Janowitz, D.; Doshi, J.; Auwera, S. Van Der; et al. White matter hyperintensities and imaging patterns of brain ageing in the general population. **2016**, 1164–1179, doi:10.1093/brain/aww008.
44. Jeerakathil, T.; Wolf, P.A.; Beiser, A.; Massaro, J.; Seshadri, S.; D'Agostino, R.B.; DeCarli, C. Stroke risk profile predicts white matter hyperintensity volume-The Framingham Study. *Stroke* **2004**, *35*, 1857–1861, doi:10.1161/01.Str.0000135226.53499.85.
45. Kern, K.C.; Wright, C.B.; Bergfield, K.L.; Fitzhugh, M.C.; Sacco, R.L.; Stern, Y.; Decarli, C.S.; Alexander, G.E. Blood Pressure Control in Aging Predicts Cerebral Atrophy Related to Small-Vessel White Matter Lesions. **2017**, *9*, 1–10, doi:10.3389/fnagi.2017.00132.
46. Kobuch, S.; Fatouleh, R.H.; Macefield, J.M.; Henderson, L.A.; Macefield, V.G. Differences in regional grey matter volume of the brain are related to mean blood pressure and muscle sympathetic nerve activity in normotensive humans. *J. Hypertens.* **2020**, *38*, 303–313, doi:10.1097/HJH.0000000000002243.
47. Mahinrad, S.; Kurian, S.; Garner, C.R.; Sedaghat, S.; Nemeth, A.J.; Moscufo, N.; Higgins, J.P.; Jacobs, D.R.;

Hausdorff, J.M.; Lloyd-Jones, D.M.; et al. Cumulative Blood Pressure Exposure During Young Adulthood and Mobility and Cognitive Function in Midlife. *Circulation* **2019**, doi:10.1161/circulationaha.119.042502.

48. Muller, M.; Sigurdsson, S.; Kjartansson, O.; Gunnarsdottir, I.; Thorsdottir, I.; Harris, T.B.; van Buchem, M.; Gudnason, V.; Launer, L.J. Late-life brain volume: A life-course approach. The AGES-Reykjavik study. *Neurobiol. Aging* **2016**, *41*, 86–92, doi:10.1016/j.neurobiolaging.2016.02.012.
49. Power, M.C.; Schneider, A.L.C.; Wruck, L.; Griswold, M.; Coker, L.H.; Alonso, A.; Jack, C.R.; Knopman, D.; Mosley, T.H.; Gottesman, R.F. Life-course blood pressure in relation to brain volumes. *Alzheimer's Dement.* **2016**, doi:10.1016/j.jalz.2016.03.012.
50. Schaare, H.L.; Kharabian Masouleh, S.; Beyer, F.; Kumral, D.; Uhlig, M.; Reinelt, J.D.; Reiter, A.M.F.; Lampe, L.; Babayan, A.; Erbey, M.; et al. Association of peripheral blood pressure with gray matter volume in 19- to 40-year-old adults. *Neurology* **2019**, *92*, E758–E773, doi:10.1212/WNL.0000000000006947.
51. Spartano, N.L.; Himali, J.J.; Beiser, A.S.; Lewis, G.D.; DeCarli, C.; Vasan, R.S.; Seshadri, S. Midlife exercise blood pressure, heart rate, and fitness relate to brain volume 2 decades later. *Neurology* **2016**, *86*, 1313–1319, doi:10.1212/wnl.0000000000002415.
52. Taki, Y.; Goto, R.; Evans, A.; Zijdenbos, A.; Neelin, P.; Lerch, J.; Sato, K.; Ono, S.; Kinomura, S.; Nakagawa, M.; et al. Voxel-based morphometry of human brain with age and cerebrovascular risk factors. *Neurobiol. Aging* **2004**, *25*, 455–463, doi:10.1016/j.neurobiolaging.2003.09.002.
53. Taki, Y.; Thyreau, B.; Kinomura, S.; Sato, K.; Goto, R.; Wu, K.; Kawashima, R.; Fukuda, H. A longitudinal study of age- and gender-related annual rate of volume changes in regional gray matter in healthy adults. *Hum. Brain Mapp.* **2013**, *34*, 2292–2301, doi:10.1002/hbm.22067.
54. Verhaaren, B.F.J.; Vernooij, M.W.; De Boer, R.; Hofman, A.; Niessen, W.J.; Van Der Lugt, A.; Ikram, M.A. High blood pressure and cerebral white matter lesion progression in the general population. *Hypertension* **2013**, doi:10.1161/HYPERTENSIONAHA.111.00430.
